# Quasi-Periodic Patterns of Neural Activity improve Classification of Alzheimer’s Disease in Mice

**DOI:** 10.1038/s41598-018-28237-9

**Published:** 2018-07-03

**Authors:** Michaël E. Belloy, Disha Shah, Anzar Abbas, Amrit Kashyap, Steffen Roßner, Annemie Van der Linden, Shella D. Keilholz, Georgios A. Keliris, Marleen Verhoye

**Affiliations:** 1 Department of Pharmaceutical, Veterinary and Biomedical Sciences, Bio-Imaging Lab, University of Antwerp, Universiteitsplein 1, 2610 Wilrijk, Antwerp Belgium; 2 Department of Biomedical Engineering, Emory University, 1760 Haygood Dr. NE, Atlanta, GA 30322 USA; 3 Department of Neuroscience, Emory University, 1760 Haygood Dr. NE, Atlanta, GA 30322 USA; 4 Department of Biomedical Engineering, Emory University and Georgia Institute of Technology, 1760 Haygood Dr. NE, Atlanta, GA 30322 USA; 5 Paul Flechsig Institute for Brain Research, University of Leipzig, Liebigstraße 19. Haus C, 04103 Leipzig, Germany

**Keywords:** Diagnostic markers, Alzheimer's disease

## Abstract

Resting state (rs)fMRI allows measurement of brain functional connectivity and has identified default mode (DMN) and task positive (TPN) network disruptions as promising biomarkers for Alzheimer’s disease (AD). Quasi-periodic patterns (QPPs) of neural activity describe recurring spatiotemporal patterns that display DMN with TPN anti-correlation. We reasoned that QPPs could provide new insights into AD network dysfunction and improve disease diagnosis. We therefore used rsfMRI to investigate QPPs in old TG2576 mice, a model of amyloidosis, and age-matched controls. Multiple QPPs were determined and compared across groups. Using linear regression, we removed their contribution from the functional scans and assessed how they reflected functional connectivity. Lastly, we used elastic net regression to determine if QPPs improved disease classification. We present three prominent findings: (**1**) Compared to controls, TG2576 mice were marked by opposing neural dynamics in which DMN areas were anti-correlated and displayed diminished anti-correlation with the TPN. (**2**) QPPs reflected lowered DMN functional connectivity in TG2576 mice and revealed significantly decreased DMN-TPN anti-correlations. (**3**) QPP-derived measures significantly improved classification compared to conventional functional connectivity measures. Altogether, our findings provide insight into the neural dynamics of aberrant network connectivity in AD and indicate that QPPs might serve as a translational diagnostic tool.

## Introduction

Alzheimer’s disease is a neurodegenerative disorder, marked by the accumulation of extracellular amyloid-beta (Aβ) plaques and intracellular neurofibrillary tangles^1^. According to the amyloid cascade hypothesis, Aβ plaques form through aggregation of soluble amyloid and plaque deposition represents the disease’s central event^2^. Alzheimer’s pathology causes the progressive loss of memory and cognitive function^3^. These could be the reflection of brain network dysfunctions^4^.

Resting state functional MRI (rsfMRI) allows the non-invasive imaging of brain networks in subjects at rest, by measurement of the blood oxygenation-level-dependent (BOLD) signal^5^. Temporal correlation of the BOLD signal across spatially distinct brain areas reveals their functional connectivity (FC). In humans, rsfMRI identifies several resting state networks (RSNs)^5,6^. The ‘default mode network’ (DMN) stands out for its high activity in subjects at rest and its deactivation during cognitive tasks. Another common network, which is anti-correlated to the DMN and active during cognitive tasks, is the Task positive network (TPN)^7,8^. The opposing interaction between DMN and TPN reflects their roles in balancing inwardly versus outwardly oriented cognitive processes^8–10^. Failure to suppress the DMN or increase TPN activation, during cognitive tasks, has been observed in patients with Alzheimer’s disease and was interpreted as disrupted brain function, reflected by increased difficulty to switch from rest to task conditions^11^.

Investigation of RSNs provides opportunity to study neurodegenerative diseases and develop biomarkers^12,13^. RsfMRI has been readily applied in human Alzheimer’s disease research, consistently revealing decreased FC in DMN areas^11,14^. Changes in DMN FC have been correlated with increased Aβ and were promising as a biomarker for Alzheimer’s disease^15,16^. It was also shown that DMN with TPN anti-correlation was disturbed in Alzheimer’s disease subjects, a finding that has been suggested amenable to improve biomarker sensitivity^17,18^.

In the preceding studies, FC was assumed to remain static across the scan duration. The brain’s functional organization is however naturally dynamic and by ignoring this, valuable information was potentially lost. Studies of dynamic FC have indeed allowed new insights into neural dynamics^19,20^, and provided potential new biomarkers for multiple neurological disorders^21,22^, including Alzheimer’s disease^23,24^. With the advance of dynamic (d)rsfMRI, a particularly interesting discovery was that of quasi-periodic patterns (QPPs)^25^. The most prominently observed QPPs represent recurring periods of large-scale anti-correlation between the DMN and TPN^26,27^. Multimodal experiments in rodents showed that QPPs in the BOLD signal are directly correlated to infraslow neural activity^28–34^, which is supported to be a key source of FC^35–45^. This relationship was not apparent with higher frequency neural activity, which was more correlated to dynamic measures of BOLD FC (e.g. sliding window FC). These studies, combined with the infraslow quasi-periodic nature of RSNs^46–49^, led to the hypothesis that QPPs reflect a specific type of ongoing BOLD fluctuations that together with other time-varying processes contribute to FC^19,32,34,50^. A potential source of QPPs and BOLD FC has been speculated under the form of neuromodulatory subcortical drive that can provide patterned input to specific brain areas^19,25,44,45^. Overall, studying QPPs represents an attractive new approach to investigate BOLD dynamics and their potential disruption.

While human studies are essential to translate research findings to clinical practice, studies in mice help gain fundamental insight into neurological disorders and establish new biomarkers. RsfMRI applications in healthy mice revealed similar RSNs as in humans^51,52^, and in models of Alzheimer’s disease pathology showed aberrant network FC consistent with clinical studies^53,54^. Finally, drsfMRI has recently provided the first insights into mouse dynamic FC and revealed the existence of QPPs in mice^27,55^.

In this paper, we postulated the hypothesis that QPPs would provide insight into the aberrant DMN and TPN FC observed in Alzheimer’s disease, and might thus serve as a more sensitive biomarker than conventional rsfMRI measures. To answer this question, we investigated QPPs in a single brain slice in old-age TG2576 mice, an amyloidosis model, and in age-matched controls. We determined how QPPs differed across groups, modeled how they reflected DMN and DMN-TPN FC, and determined if that information could be used to improve classification. Our findings provide new insights into the neural dynamics that reflect aberrant network connectivity in a mouse model of Alzheimer’s disease, and promise that QPPs may serve amenable as a new translational biomarker.

## Material and Methods

### Ethical statement

All procedures were performed in strict accordance with the European Directive 2010/63/EU on the protection of animals used for scientific purposes. The protocols were approved by the Committee on Animal Care and Use at the University of Antwerp, Belgium (permit number 2014–04), and all efforts were made to minimize animal suffering.

### Animals

The TG2576 mouse model of amyloidosis overexpresses the human mutant form of amyloid precursor protein (APP), which carries the Swedish mutation (KM670/671NL), controlled by the hamster prion protein promoter^56^. Aβ plaque development starts at the age of 9–11 months^56^, while plaque burden increases markedly with age^57^. In the current study, rsfMRI was acquired in female TG2576 mice at the age of 18 months (TG, N = 10) and in age-matched wild-type littermates (WT, N = 8). For rsfMRI recordings, animals were sedated using 0.4% isoflurane, a bolus injection of medetomidine (0.3 mg/kg), and a subcutaneous infusion of medetomidine (0.6 mg/kg/h). Additional details on animal handling are provided in Supplementary Methods.

### MRI procedures and functional scan pre-processing

MRI scans were acquired on a 9.4 T Biospec system, with a four-element receive-only phase array coil and a volume resonator for transmission. Structural images were acquired in three orthogonal directions, using Turbo Rapid Imaging with Refocused echoes (RARE), for reproducible slice positioning (repetition time 3000 ms, effective echo time 33 ms, 16 slices of 0.4 mm). B0 field maps were acquired, followed by local shimming. RsfMRI scans were acquired with a gradient-echo echo-planar imaging (EPI) sequence (field of view (20 × 20) mm^2^, matrix dimensions [128 × 64], three slices of 0.4 mm, flip angle 55°, bandwidth 400 kHz, repetition time 500 ms, echo time 16 ms, 2400 repetitions). High temporal resolution was required to investigate QPPs. Due to resultant technical limitations, slice number was restricted to three. Slices were positioned 0.1 mm caudally of bregma, according to the Paxinos and Franklink stereotaxic mouse brain atlas^58^.

Motion parameters for each functional scan were obtained using 6 rigid body parameters. Images were realigned and normalized to a user-defined reference subject, followed by smoothing (σ = 2 pixels). During image normalization, intensities of outer slices are lost. Analyses were thus restricted to the single center slice (MATLAB2017b). Motion vectors were then regressed out of the image series. These procedures were performed using Statistical Parametric Mapping (SPM12) software (Wellcome Department of Cognitive Neurology, London, UK). Images were then filtered using a 0.01–0.2 Hz FIR band-pass filter, quadratic detrended and normalized to unit variance. Transient time points at the start and end of the image series were removed before and after filtering. For the detection of spatiotemporal patterns, a brain mask was employed to exclude contribution of the ventricles. QPPs were obtained without performing global signal regression, unless otherwise stated.

### Detection of quasi-periodic patterns

We identified QPPs in both WT and TG animals, using a spatiotemporal pattern finding algorithm^25^. QPPs were determined by applying the algorithm to a group image series, composed of the center-slice concatenated scans of individual subjects. In brief, the algorithm identifies sets of consecutive images, i.e. spatiotemporal patterns, which recur within a functional scan. First, a template, that is, a set of images acquired at directly consecutive time points, is selected at a random time point in the image series. The user defines the number of images within the template, which is termed the. the window length. The template is compared to the group image series via sliding correlation, at each time point, i.e. the template is incrementally shifted one image frame at a time. The resultant correlation time series is termed the **S**liding **T**emplate **C**orrelation (STC). Peak threshold crossings, above an arbitrary correlation value (0.2), are used to identify sets of consecutive images that will be averaged into a new updated template. The 0.2 threshold is a heuristic value, consistent with prior work on QPPs^25–27^. This sliding correlation process is repeated until convergence of the spatiotemporal pattern from iteration to iteration. This identifies a single QPP, at a user-defined window length and for a single random starting time point. The random starting time point affects the final outcome, so the process is repeated multiple times (N = 500). The entire process is repeated for multiple window lengths.

### Quasi-periodic pattern spatial configurations and temporal lengths

Visual inspection suggested that short QPPs displayed opposing intensities between neuroanatomical regions, while longer QPPs involved multiple brain regions simultaneously. To quantify this observation, we calculated the mean coefficient of variation (C_V_) of QPP image intensities across time. Specifically, QPPs describe a single brain slice, measuring m by n voxels, across multiple time points (T), and are therefore represented by a 3D-matrix. This slice can also be described under a vector form, so that QPPs can be formulated as the L by T matrix QPP2D, where L = m × n. Mean C_V_ was determined as $\overline{{\rm{QPPcv}}}$ = $\sum _{t=1}^{T}|\overline{C{v}_{t}}|/{\rm{T}}$, where T is the number of image volumes in the QPP, and $\overline{C{v}_{t}}$ = σ (QPP2D_T_)/μ (QPP2D_T_), where μ is the mean and σ the standard deviation. A high $\overline{{\rm{QPP}}Cv}$ indicates that slices within the QPP displayed opposing intensities, and therefore high contrast.

Measures of QPP contrast were used to guide identification of interesting window sizes. When a similar QPP type was observed across different window sizes of analysis, a previously established analysis strategy was employed to determine its true window length^27^ (cfr. Supplementary Methods).

### Selection of quasi-periodic patterns

At each investigated window size, in each group, a single QPP was selected that displayed the highest sum of peak correlation values, exceeding the 0.2 correlation threshold, in its STC. This procedure matches a recently established criterion for QPP selection^26^.

### Comparing quasi-periodic patterns across groups

**Figure 1:**
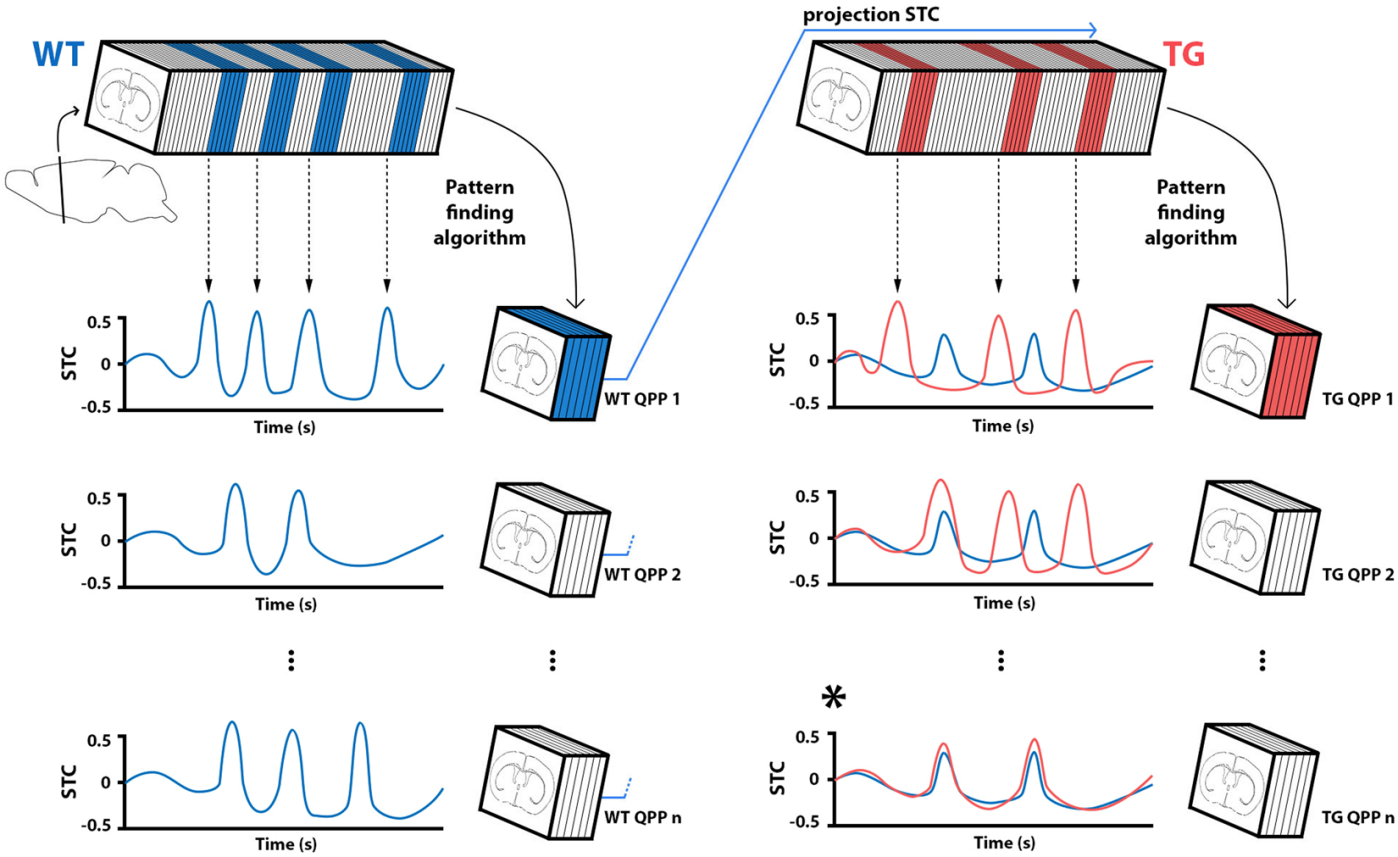
Group comparisons of Quasi-Periodic patterns via ‘projection’. QPPs were independently derived from the WT (left, blue) and TG (right, red) group, using a spatiotemporal pattern finding algorithm^25^. In each group, the algorithm was used to obtain a set of n QPPs at each investigated window length (illustrative QPPs are displayed on the three lower rows). Each of these QPPs is accompanied by a Sliding Template Correlation (STC) series, i.e. the sliding correlation of the QPP with the functional image series from which it was derived (color-coded traces next to respective QPPs). Peak correlations above an arbitrary threshold indicate QPP occurrences. This is illustrated for top row QPPs (arrows), indicating corresponding images in the functional scans. QPPs derived from one group’s image series (reference), can be compared with the other group’s image series (target) via projection (p)STC (e.g. blue arrow from WT QPP1 onto TG image series). Occurrences of one group’s QPPs can thus be determined in the opposing group. Additionally, pSTCs from a reference QPP can be compared with STCs from QPPs in the target group (blue and red traces in right panels). The goal of this comparison is to determine the closest matching QPP, which correlates to the image series in the same way. pSTCs and STCs with the strongest similarity, determined via cross-correlation, identify corresponding QPPs across groups (*). Schematic brain images indicate the brain slice investigated in the current study. *Abbreviations*. *QPP*, *Quasi*-*Periodic Pattern*; *pSTC*, *projection sliding*-*template correlation*.

Each individual QPP is accompanied by a STC with the image series from which it was derived. This STC describes the similarity of the QPP with the image series and identifies its occurrences, defined as the amount of STC peaks exceeding a 0.2 correlation threshold. After QPPs are extracted from a particular dataset, STCs to another image series can also be constructed (Fig. 1), a measure that we termed ‘projection STC’ (pSTC). This allowed occurrences of a QPP to be directly compared across groups. Additionally, QPPs that were independently identified in a respective group could be matched with those of the other group.

### Removing quasi-periodic patterns

Individual QPPs were convolved with their respective STC to construct QPP image series. The obtained QPP image series were linearly regressed voxel-wise out of the original scans, removing their contribution to the BOLD signal. The residual image series after regression were used to assess the impact of QPPs on FC.

### Functional connectivity analysis

ROIs were selected matching a stereotaxic mouse brain atlas^58^ and used to construct voxel-wise seed-based FC-maps and ROI-based FC-matrices for each subject. FC-values were Fisher Z-transformed (zFC) to allow group comparisons. To investigate DMN- and DMN-TPN-like FC, zFC-values between ROI pairs within the portion of these networks, present in the investigated slice, were averaged per subject. TPN-like ROIs included Insular cortex (Ins), ventro-lateral Caudate Putamen (Cpu vl), somatosensory area 1 (S1) and area 2 (S2). DMN-like ROIs included Cingulate cortex (Cg) and dorsal Cpu (d).

### Quasi-periodic pattern inter-area correlation

QPPs’ inter-area relationship was determined by averaging ROI-pair Pearson correlations ($\bar{{\rm{\rho }}}$).

### Classification analysis

A total of 17 rsfMRI measures were independently used as predictors in elastic net logistic regression models, to classify mice as either WT or TG. Additionally, all 17 measures were used simultaneously in a combined model. Elastic net regression is a regularisation technique for general linear models and is particularly useful in that predictor coefficients may be reduced to zero in case of poor fitting. It has been commonly used for classification purposes in neuroimaging studies and classification of Alzheimer’s disease^24,59–64^. To evaluate classification performance, we constructed receiver operating characteristic (ROC) curves and calculated the area under the curve (AUC). Cross-validation of each model was repeated 10 times to obtain reliable cross-validation errors and determine mean AUC-values. Additional details are provided in Supplementary Methods.

### Statistical analyses

Significant voxels within a QPP were determined from the voxel-wise intensity distribution of unique image frames averaged to establish QPPs (one-sample T-test, two-tailed, FDR p < 0.001). QPP occurrence rates were determined per subject in the respective group, and using pSTC (Fig. 1), in the other group. Each QPP’s occurrence rates were compared across animals between WT and TG (two-sample T-test, two-tailed, p < 0.05).

For within-group statistical analysis of seed-based FC maps, one sample T-tests (two-tailed, uncorrected p < 0.05) were performed. For between-group statistical analysis of seed-based FC maps, before and after QPP regression, paired T-tests (uncorrected p < 0.05) were performed. For within group statistical analysis of DMN-like and DMN-TPN-like FC, before and after QPP regression, paired T-tests (uncorrected) were performed. For between group statistical analysis of DMN-like and DMN-TPN-like FC, comparing WT with TG, two-sample T-tests (two-tailed, uncorrected) were performed.

To statistically compare between AUC-values of the investigated classification models, AUC-values were bootstrapped 2000 times, with stratification, for each pair^65,66^. Models were compared with two-sided tests. P-values are provided uncorrected and after Bonferroni correction.

## Results

### General findings and quasi-periodic pattern selection criteria

In both groups, 3 s QPPs displayed high spatial contrast, i.e. regional anti-correlation, and similar occurrence rates across groups, while longer QPPs displayed low spatial contrast, i.e. global signal-like structure involving many brain areas, and higher occurrence rates in WT compared to TG (Supplementary Fig. S1A–D). At each window size, the QPPs that most strongly correlated with the image series confirmed this observation (Supplementary Fig. S1I–J). Global signal-like QPPs displayed consistent spatiotemporal profiles across window sizes, and shared similarity between WT and TG. For each group, these QPPs were considered as a single type, and their true window lengths were determined to be 6 s (Supplementary Fig. S2).

3 s QPPs were visually inspected to scope the richness of spatiotemporal patterns and were compared with RSNs, determined with independent component analysis (ICA) (Supplementary Fig. S3 and Supplementary Methods). ICA is a commonly used analysis method in rsfMRI to obtain maximally independent components that are present within the data. The outcome is a set of ‘static’ spatial maps, i.e. RSNs, and related time vectors that indicate their similarity to the image series over time. The latter is similar to the outcome of QPP analysis, but the difference is that QPPs capture the spatiotemporal evolution of network activity over time. Supplementary Fig. S3 indicates that ICA-derived RSNs appeared similar to some QPPs, but that a wider range of spatiotemporal patterns could be observed in QPPs, indicating that this new analytical approach can provide additional insight into the data.

Supplementary Fig. S3 also illustrates that 3 s QPPs could be observed in opposite phases (inverted activity profiles). By averaging additional image frames outside of the 3 s window size, it was confirmed that 3 s QPPs are part of larger bi-phasic QPPs, consistent with prior work^27^, but that these are less reliably detected in the current study (Supplementary Fig. S4). If global signal regression was performed, all QPPs displayed high spatial contrast, but occurrence rates dropped at window sizes longer than 3 s (Supplementary Fig. S1E–H). Both findings indicate that bi-phasic high contrast QPPs were not reliably observed (cfr. discussion). Further analyses were thus restricted to 3 s high contrast and 6 s low contrast QPPs.

### Differences in Quasi-Periodic brain activity between wild-type and TG2576 mice

**Figure 2:**
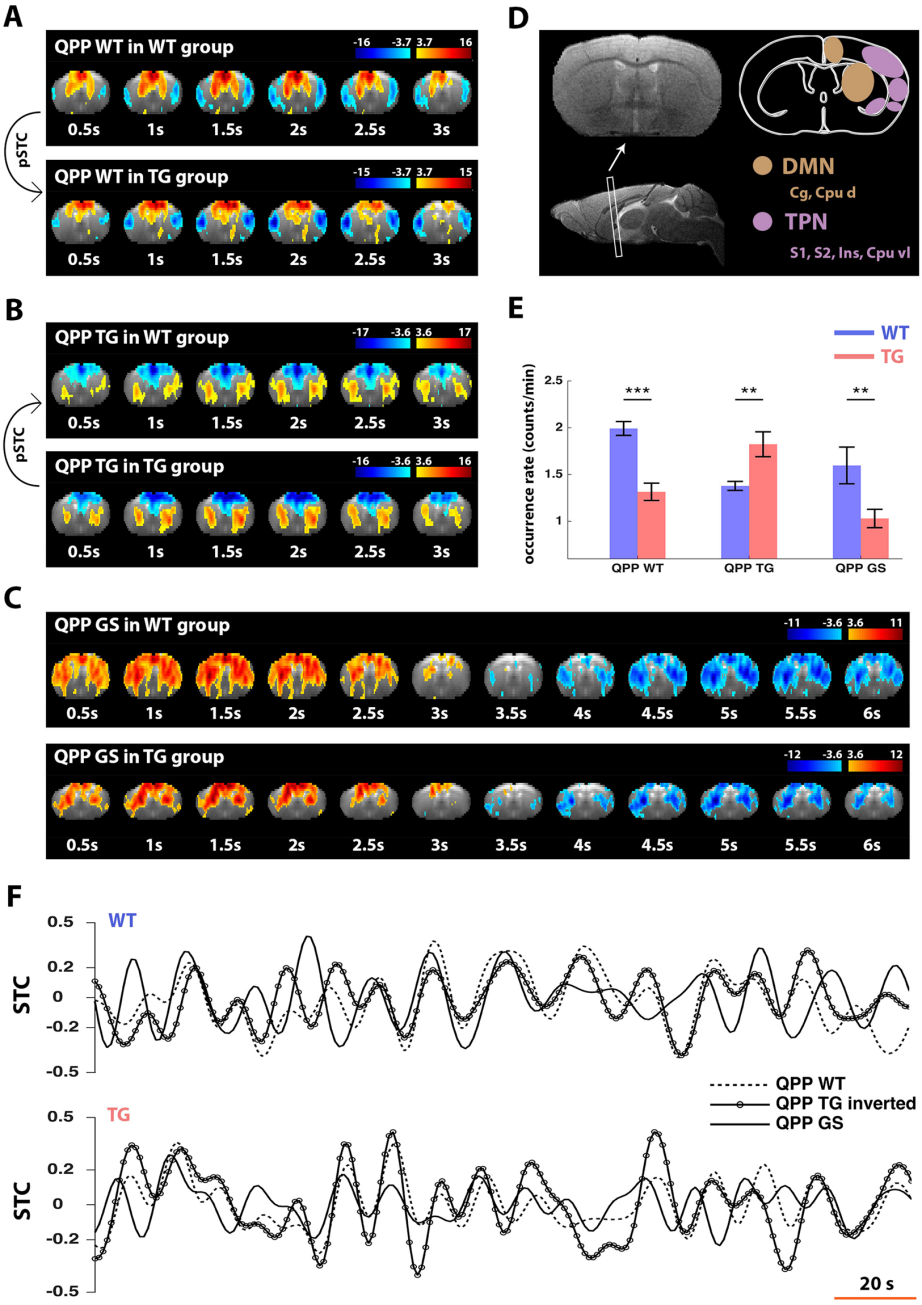
Three Quasi-Periodic patterns indicate the largest group differences in spatiotemporal brain dynamics. (**A**–**C**) The QPPs that most strongly correlated with the image series in WT and TG, at 3 and 6 s window sizes, are shown. Because 3 s QPPs displayed highly different patterns across groups, pSTC was used to determine the closest matching QPP in the opposing group. QPPs are displayed as thresholded T-maps, overlain on the respective brain image across time (one-sample T-test, FDR p < 0.001). (**D**) Location of the investigated brain slice and anatomical regions that correspond to hypothesized regions of the mouse DMN- and TPN-like networks. (**A**) The 3 s QPP derived from the WT group (QPP WT). Note in red, co-activity of Cg and Cpu d areas (DMN). In blue, de-activated areas are S1, S2, Ins and Cpu vl (TPN). The matching QPP in TG displayed a similar pattern, with less ‘intact’ DMN- and TPN-like networks. (**B**) The 3 s QPP derived from the TG group (QPP TG). Note, as opposed to QPP WT, that QPP TG displayed anti-correlation between Cg and Cpu d regions. The matching QPP in WT displayed a similar pattern, which included co-activation of some TPN-like components. (**C**) The 6 s global signal-like QPP derived from the WT (top) and TG (bottom) groups. Both displayed a similar spatiotemporal shape (spatial correlation = 0.75). This QPP is commonly referred to as QPP GS. Note the involvement of many brain regions. In TG, lateral cortical areas (TPN-like) appeared less clearly co-active. (**E**) Subject mean occurrence rates for all QPPs, based on STC and pSTC (two-sample T-test, **p < 0.01, ***p < 0.001, error flags show standard error). (**F**) Illustration of temporal overlap between the three described QPPs in their respective groups. The STC of QPP TG was inverted to account for its opposite phase. *Abbreviations*. *FDR*, *False*-*Discovery Rate*; *GS*; *global signal*; *STC*, *sliding*-*template correlation series*; *cc*, *cross*-*correlation*; *Cg*, *Cingulate area*; *Cpu d*, *Caudate Putamen dorsal*; *S*1, *somatosensory area 1*; *S*2, *somatosensory area* 2, *Cpu vl*, *Caudate Putamen ventro*-*lateral*; *Ins*, *Insular cortex*; *Pir*, *piriform cortex*; *DMN*, *Default Mode network*; *TPN*, *Task*-*Positive network*.

The QPPs that most strongly correlated to each group’s image series indicated functional differences between WT and TG. The 3 s QPP from the WT group (QPP WT) displayed co-activity between Cpu d and Cg areas ($\bar{{\rm{\rho }}}$ = 0.95) (Fig. 2A). These are established components of the mouse DMN-like network^51,52,67^. Within QPP WT, the DMN-like areas were anti-correlated with S2, S1, Ins and Cpu vl (respectively $\bar{{\rm{\rho }}}$ = −0.73, −0.36, −0.64, −0.69). These are part of the lateral cortical network, speculated to represent a mouse TPN-like network^51^ (Fig. 2D). Using pSTC (Fig. 1), it was determined that QPP WT occurred significantly less in TG mice (Fig. 2E), and that the closest matching QPP in TG mice displayed diminished anti-correlation between the DMN-like network and S2, S1, Ins and Cpu vl (respectively $\bar{{\rm{\rho }}}$ = −0.52, 0.21, 0.07, −0.17). The 3 s QPP from the TG group (QPP TG) displayed co-activity between Cpu d areas (ρ = 0.98), which were anti-correlated with Cg ($\bar{{\rm{\rho }}}$ = −0.97) (Fig. 2B). There appeared little anti-correlation of Cg with S2, S1, Ins and Cpu vl (respectively $\bar{{\rm{\rho }}}$ = 0.06, 0.32, −0.04, −0.91). Via pSTC, it was determined that QPP TG occurred significantly more in TG versus WT (Fig. 2E). The closest matching QPP in the WT group displayed less anti-correlation between Cg and Cpu d ($\bar{{\rm{\rho }}}$ = −0.52), and more anti-correlation between Cg, and S2, S1, Ins and Cpu vl (respectively $\bar{{\rm{\rho }}}$ = −0.69, −0.12, −0.63, −0.82). Lastly, a 6 s biphasic global signal-like QPP was derived from both groups. In the WT group, S2, S1, Ins and Cpu vl were correlated with DMN-like areas (respectively $\bar{{\rm{\rho }}}$ = 0.92, 0.97, 0.76, 0.78) (Fig. 2C). In the TG group, S2, S1, Ins and Cpu were less strongly correlated with DMN-like areas (respectively $\bar{{\rm{\rho }}}$ = 0.58, 0.48, 0.54, 0.61). The global signal-like QPPs in WT and TG displayed strong overlap with the brain global signal (respectively STC cross-correlation: 0.70 and 0.71). While both QPPs displayed some differences in their spatiotemporal profiles, they did share an overall similarity (spatial correlation: 0.74). We refer to this QPP commonly as ‘QPP GS’, reasoning it reflects the same QPP, with lowered spatial integrity and occurrence in TG (Fig. 2E).

Noticeably, QPP WT and QPP TG displayed temporal overlap with one another (STC cross-correlation: in WT 0.74 and TG 0.63, temporal lags = 0 s) (Fig. 2F). To calculate this cross-correlation, the STC of QPP TG was inverted to account for the opposite phase of DMN-like activity compared to QPP WT. This procedure is sensible given the biphasic nature of QPPs (Supplementary Figs S3 and 4). Both QPPs also displayed temporal overlap with QPP GS (STC cross-correlation: QPP WT - in WT 0.27 and in TG 0.34; QPP TG - in WT 0.24 and in TG 0.43; all temporal lags <1.5 s).

### Quasi-Periodic patterns reflect functional connectivity group differences

**Figure 3:**
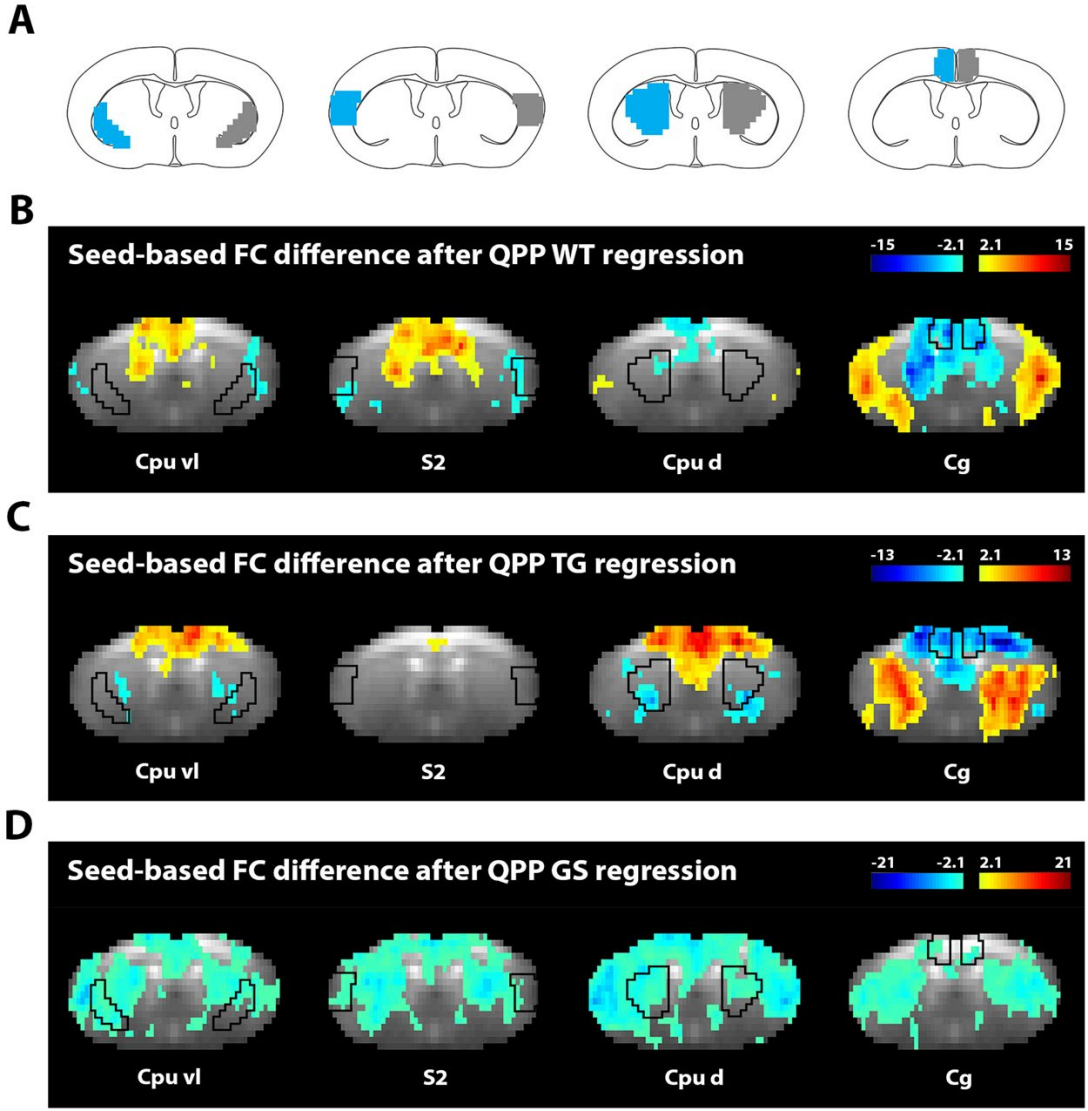
Regression of Quasi-Periodic patterns reveals how they reflect functional connectivity. QPPs are hypothesized to reflect a time-varying process that contributes to BOLD FC. To confirm this hypothesis, we investigated the change in seed-based FC after linear regression of each QPP out of the image series. (**A**) Left seed regions, and their contralateral counterpart, are indicated in respectively blue and grey on representative schematic brain images. (**B**–**D**) FC difference maps for left seed regions, specified at the bottom of the panel, after regression of QPP WT in WT group (**B**) QPP TG in TG group (**C**) and QPP GS in WT group (**D**). Seed locations and contralateral counterparts are indicated by black contours. Maps show T-values, based on voxel-wise zFC distributions of all animals in the respective group, and are liberally thresholded to show the full extent of FC change (two-sample T-test, p < 0.05). Positive and negative T-values display voxels that, after QPP regression, respectively demonstrated increased or decreased FC with respect to the seed. Note for all three QPPs the loss of FC between brain regions that were co-active within the QPP, and the increase in FC between areas that were anti-correlated. *Abbreviations*. *Cg*, *Cingulate area*; *Cpu d*, *Caudate Putamen dorsal*; *S2*, *somatosensory area 2*, *Cpu vl*, *Caudate Putamen ventro*-*lateral*.

Prior work suggested QPPs represent a specific time-varying process that reflects large-scale FC^19,28,32–34,50^. Particularly, it was proposed that QPPs are dissociable from other types of time-varying BOLD activity. In line with this hypothesis, we linearly regressed QPPs out of the functional image series of their respective groups, removing their contribution (Supplementary Fig. S5A). While there are limitations to using a linear model approach (cfr. discussion), we employed this framework to evaluate FC differences before and after QPP regression, in order to model how QPPs may reflect FC. Seed-based FC maps are presented in Supplementary Fig. S5B–D, confirming bilateral network FC before QPP regression. Seed-based FC T-contrast maps indicate voxel-wise FC-changes with known neuroanatomical locations (Fig. 3). Overall, QPP regression indicated decreased FC between regions that were co-active within the QPPs, and increased FC between regions that were anti-correlated. Per illustration, after QPP WT regression, Cg and S2 no longer displayed bi-lateral FC, but FC between them was increased. These are respectively DMN- and TPN-like areas.

**Figure 4:**
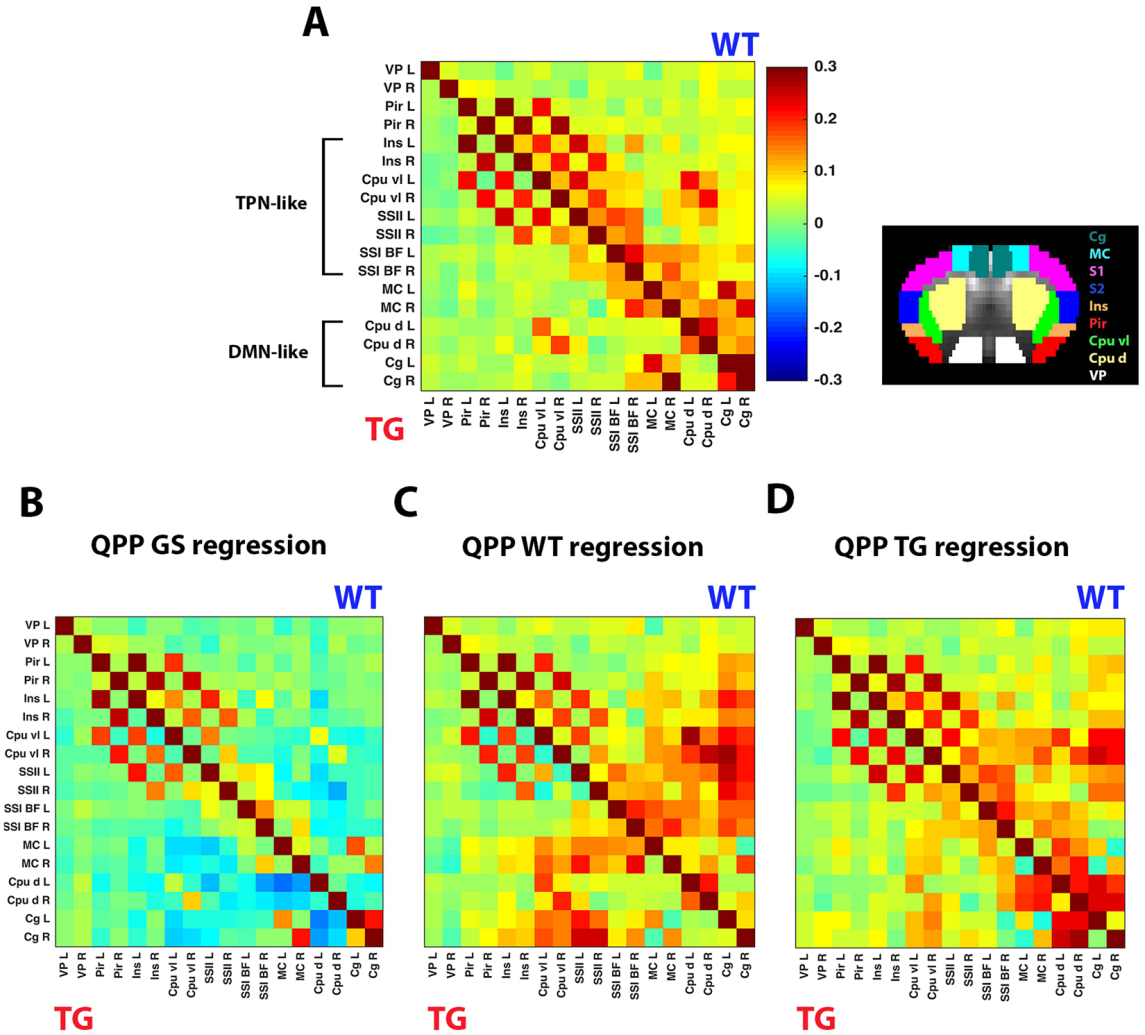
Regression of Quasi-Periodic patterns reveals how they reflect functional connectivity differences between wild type and transgenic mice. (**A**) ROI-based zFC matrix showing in the top triangle subject-average values for the WT group and in the lower triangle values for the TG group. ROI-locations are indicated on the right. Note lower DMN-like FC in TG compared to WT. (**B**–**D**) Same as in (**A**) but now after first regressing in both groups: (**B**) QPP GS, (**C**) QPP WT, and (**D**) QPP TG. ROI FC matrices indicate that QPP regression affected DMN-like (Cpu d and Cg) FC differences by either (**B**) further emphasizing the difference and diminishing FC, (**C**) removing the difference and diminishing FC, or (**D**) removing the difference and increasing FC. Another major effect of QPP regression was the increased correlation between DMN-TPN-like brain areas, hinting new group differences.

QPPs displayed interesting opposing brain dynamics between WT and TG (Fig. 2). After modelling how QPPs relate to FC (Fig. 3), we wanted to assess if they reflect FC differences between WT and TG. First, a ROI-based FC matrix was constructed to compare WT with TG (Fig. 4A). In TG animals, mostly the DMN-like areas Cg and Cpu d displayed decreased FC. The same FC matrices were then constructed after one-by-one regression of each of the investigated QPPs in their respective groups (cfr. Fig. 2) to assess resultant FC changes (Fig. 4B–D). Regression of QPP GS caused an overall FC decrease in both WT and TG (Fig. 4B). Regression of QPP WT caused a FC increase between DMN- and TPN-like areas (Fig. 4C). This effect appeared stronger in WT versus TG. DMN-like FC was lowered and no longer appeared different between both groups, suggesting that QPP WT reflected DMN-like FC group differences. Regression of QPP TG caused a less pronounced FC increase between DMN- and TPN-like areas in WT, and even less so in TG (Fig. 4D). In contradiction to QPP WT regression, DMN-like FC was increased, while its differences across groups appeared diminished.

### Quasi-periodic patterns reflect decreased Default mode like network functional connectivity in transgenic mice

**Figure 5:**
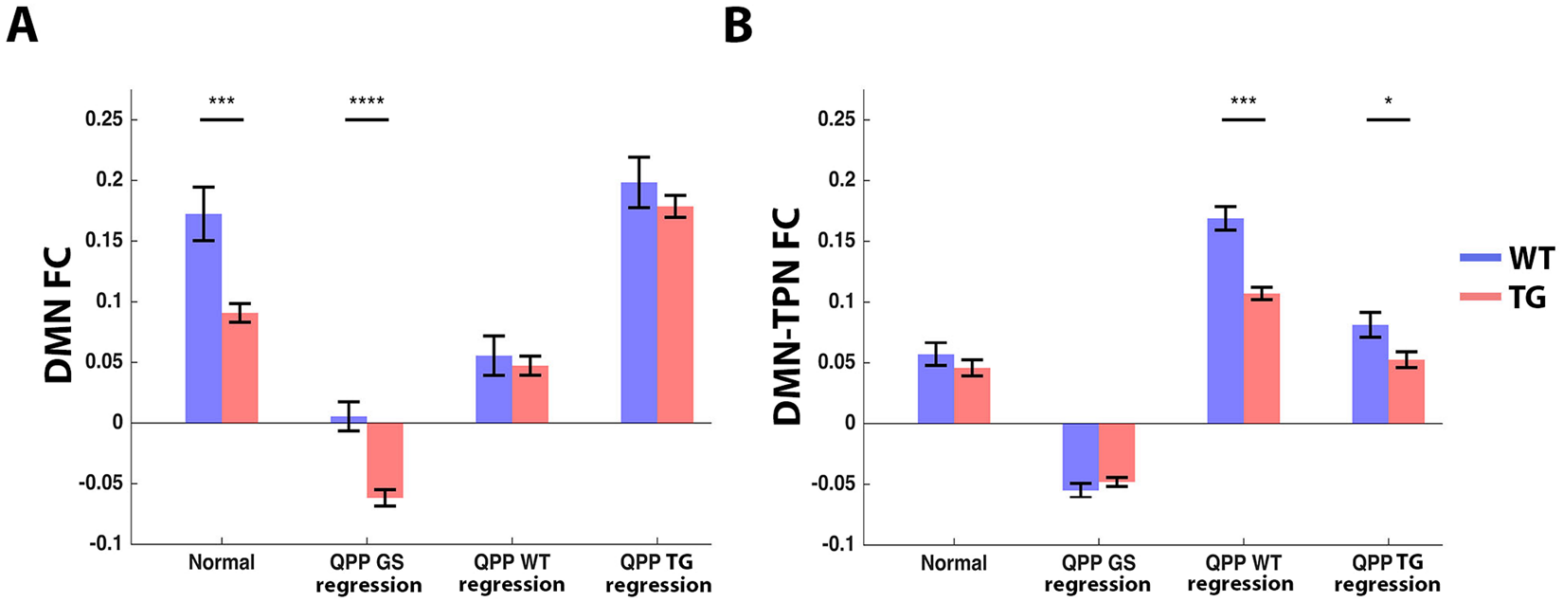
Quasi-Periodic patterns reflect default mode network functional connectivity group differences, and reveal decreased default mode with task positive network dynamic anti-correlation in transgenic mice. Subject average zFC values between (**A**) all DMN-like area pairs and (**B**) all DMN-TPN-like area pairs. Bar graphs display group average FC under conditions of no QPP regression (left) and after regression of indicated QPPs (two-sample T-test, *p < 0.05, ***p < 0.001, ****p < 0.0001, error flags indicate standard error). (**A**) Note how QPP GS regression increased the significance of the DMN-like FC group difference. Regression of QPP WT, which showed clear involvement of DMN-like areas, lowered overall DMN-like FC and removed significant WT-TG differences. Regression of QPP TG, which showed anti-correlation between DMN-like areas, increased overall DMN-like FC and removed WT-TG differences. Thus, QPPs in WT and TG displayed opposing brain dynamics, which appeared to reflect DMN-like FC differences between groups. (**B**) Regression of QPP TG, and especially QPP WT, which showed anti-correlation between DMN-TPN-like regions, revealed DMN-TPN-like FC increases that were significantly higher for WT compared to TG animals. Thus, QPPs appeared to be responsible for anti-correlated DMN-TPN-like dynamics, which could not be identified with conventional rsfMRI analysis.

Results indicated that QPP regression altered FC between DMN-like areas (Fig. 4). QPPs may therefore reflect lowered DMN-like FC in TG mice and may serve to improve sensitivity to this readout. To address this hypothesis, FC between all Cg and Cpu d ROI-pairs were averaged for each subject and compared across groups (Fig. 5A). Under normal conditions, DMN-like FC was significantly lowered by 47% in TG versus WT (two-sample T-test, p < 1 × 10^−3^). After regression of QPP GS, DMN-like FC was significantly lowered by 96% for WT and shifted below zero for TG (paired T-test, WT p < 1 × 10^−6^, TG p < 1 × 10^−11^). The significance of the DMN-like FC group difference was increased (two-sample T-test, p < 1 × 10^−4^), as a consequence of DMN-like FC standard error decrease in WT mice (55%). After QPP WT regression, DMN-like FC was significantly decreased by 70% in WT and by 26% in TG (paired T-test, WT p < 1 × 10^−5^, TG p < 1 × 10^−4^). After QPP TG regression, DMN-like FC significantly increased by 18% in WT and by 101% in TG (paired T-test, WT p < 1 × 10^−3^, TG p < 1 × 10^−7^). After regression of QPP WT and QPP TG, significant differences in DMN-like FC were no longer present (T-test, WT p = 0.66, TG p = 0.57).

### Quasi-Periodic patterns reveal decreased dynamic anti-correlation between the Default mode and Task positive like network in transgenic mice

FC between DMN- and TPN-like areas appeared increased by regression of QPP WT and QPP TG (Fig. 4). Given their spatiotemporal shape and modelled contribution to BOLD FC, this might reflect a removal of dynamic DMN-TPN anti-correlations, which could not be evaluated without QPP regression. To further investigate this, FC between DMN- and TPN-like areas were averaged for each subject and compared across groups (Fig. 5B). Under normal conditions, DMN-TPN-like FC was low (±0.05) in both WT and TG, with no significant group difference (two-sample T-test, p = 0.39). After regression of QPP GS, DMN-TPN-like FC values were significantly lowered below zero (paired T-test, WT p < 1 × 10^−6^, TG p < 1 × 10^−6^). No significant group difference was revealed (two-sample T-test, p = 0.29). After regression of QPP WT, DMN-TPN-like FC was significantly increased by 193% in WT and 131% in TG (paired T-test, WT p < 1 × 10^−7^, TG p < 1 × 10^−7^). The DMN-TPN-like FC increase was significantly higher in WT versus TG (two-sample T-test, p = 1 × 10^−3^). After regression of QPP TG, DMN-TPN-like FC was significantly increased by 32% for WT (paired T-test, WT p < 1 × 10^−4^) and non-significantly by 8% for TG. The DMN-TPN-like FC increase was significantly higher in WT versus TG (two-sample T-test, p = 0.02).

### Quasi-periodic pattern derived measures improve classification

**Figure 6:**
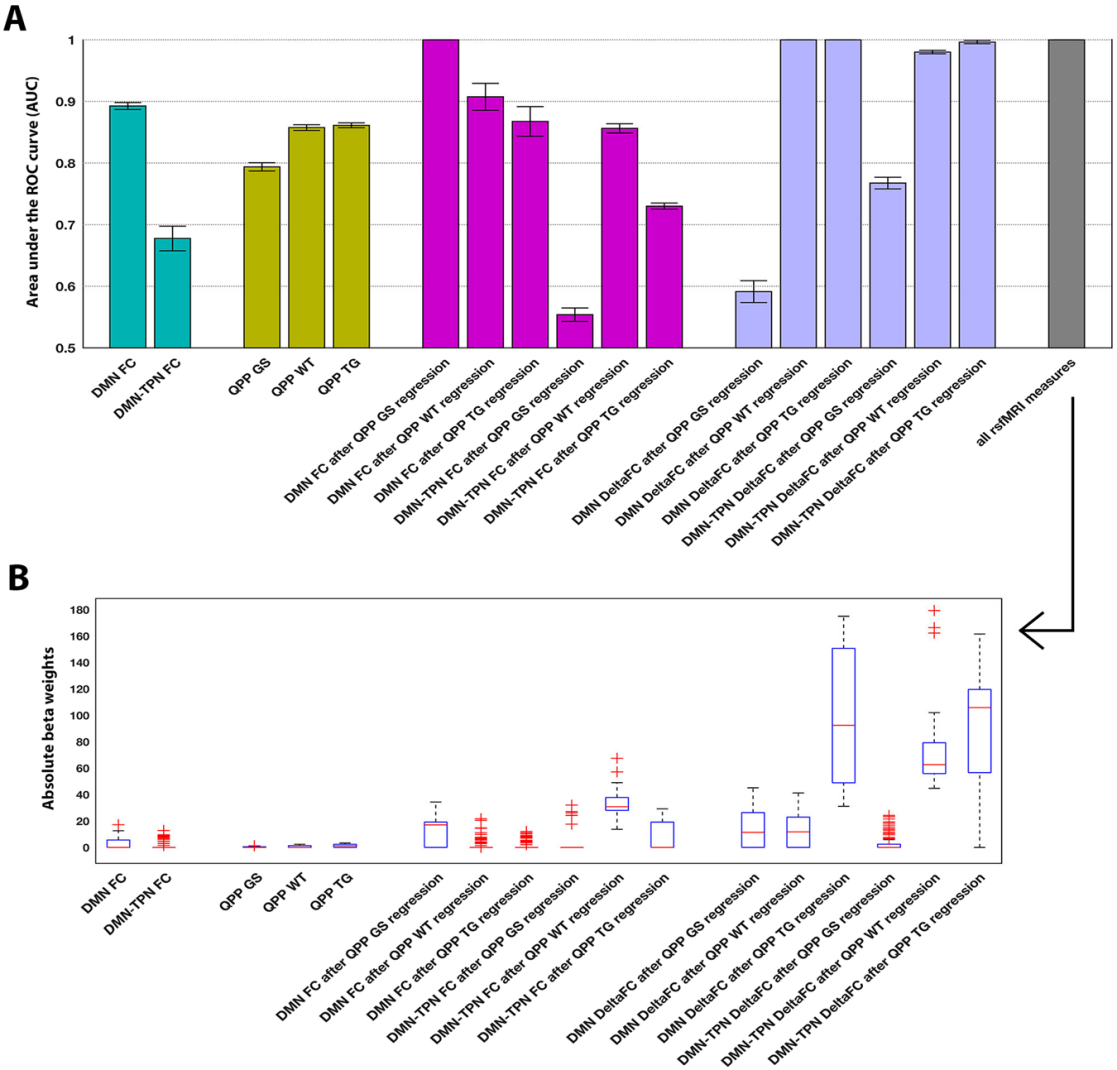
Classification is improved through Quasi-Periodic Patterns. (**A**) AUC-values of ROC-curves constructed for classifications based on each individual resting state measure and for the combined model, which evaluated all measures simultaneously (right, grey). AUC-values are shown as mean and standard error of the mean. Note 100% classification for some QPP-derived measures. (**B**) All 100 absolute beta values for each individual measure in the combined model, obtained through repeated model cross-validation. High beta-values imply the importance of the respective measure. DMN- and DMN-TPN-like ΔFC after QPP TG regression, together with DMN-TPN-like ΔFC after QPP WT regression, showed the highest contributions. Overall, findings indicate that subject-wise contribution of QPPs to FC provides the most sensitive measure for classification. *Abbreviations*. *AUC*, *Area Under the Curve*; *ROC*, *Receiver Operating Characteristic*; ΔFC, Functional connectivity change.

After determining that QPPs may provide new insight into aberrant network FC in TG mice, we wondered whether they might also improve subject classification. To answer this question, we used elastic net regression to investigate the classification performance of individual network FC measures compared to individual QPP-related rsfMRI measures. All measures were also simultaneously investigated in a combined model. The AUC-values of ROC-curves (classification performance index) for all measures are presented in Fig. 6A, while statistical comparisons are presented in Supplementary Fig. S6. DMN-like FC displayed high performance (AUC: 0.89) and TPN FC low performance (AUC: 0.68). QPP occurrence rates did not display higher performance compared to DMN-like FC (AUC: QPP GS 0.79, QPP WT 0.85, QPP TG 0.86). When QPP GS was regressed, DMN-like FC allowed full classification. Similarly, DMN-like FC differences (ΔFC) between individual subjects, before and after QPP WT or QPP TG regression, allowed full classification. DMN-TPN-like ΔFC also displayed high classification performance (AUC: QPP WT 0.98, QPP TG 0.99). The combined model allowed full classification. Figure 6B displays the absolute beta weights for each measure in the combined model. High betas indicate the importance of each respective measure. DMN- and DMN-TPN-like ΔFC after QPP TG regression appeared as the strongest contributors, followed by DMN-TPN-like ΔFC after QPP WT regression. Beta weights were mostly in line with the respective AUCs for individual parameters, but stressed the importance of QPP TG regression and DMN-TPN-like ΔFC for classification.

## Discussion

In the current work, we investigated quasi-periodic patterns of BOLD activity in a transgenic mouse model of Alzheimer’s disease compared to age-matched controls. QPPs have been observed in multiple species in which they display anti-correlation between the DMN and TPN^25–27^. These resting state networks are affected in patients with Alzheimer’s disease, an observation that has been suggested amendable towards biomarker development. The neural dynamics that underlie their aberrant FC have however remained elusive and classification requires further improvement in order to support clinical utility^13,16,68^. We illustrate how in an aged mouse model of Alzheimer’s disease, QPPs provide novel insights into aberrant DMN and TPN FC and improve classification. Our work brings forward three prominent findings: (1) WT and TG mice were marked by different QPPs, that is, WT displayed significantly higher occurrences of a QPP in which DMN areas were co-active and anti-correlated with the TPN, while TG displayed significantly higher occurrences of a QPP in which DMN areas were anti-correlated. (2) We then linearly regressed QPPs from the functional scans to model how they reflect FC differences between groups. Using this analytical framework, it could be shown how QPPs reflected significantly lowered DMN FC in TG mice and additionally how they revealed a significant decrease in dynamic DMN-TPN anti-correlations, an observation that could not be made without QPPs. (3) Lastly, we show that FC-measures derived through QPP regression improved classification compared to conventional FC-measures. Altogether, our findings provide new insights into the neural dynamics that reflect aberrant network FC in a mouse model of Alzheimer’s disease and indicate that QPPs can serve as a translational tool to improve disease classification.

Resting state networks, within the single slice investigated in the current study, match those in preceding mouse literature. Our FC maps and matrices with seeds in Cpu d and Cg confirmed the interdependence of these areas, a finding consistent with observations of the DMN and its underlying monosynaptic connectivity^51,67,69^. QPP WT similarly displayed co-activation of these DMN areas. So far, the existence of a mouse TPN has only been speculated, in that a mouse lateral cortical network (LCN) comprises homologous brain areas to those in humans and displays similar anti-correlation with the DMN^51,52^. The main nodes of the mouse LCN, which we used to define the TPN, have been consistently reported in mouse rsfMRI literature^27,67,70–72^. Our FC maps with seeds in somatosensory areas revealed the LCN (Supplementary Fig. S5). QPP WT also displayed anti-correlation between this network and the DMN, an observation consistent with human QPPs and supporting the existence of a mouse TPN^25,26^. Lastly, FC maps revealed overall bilateral connectivity, which was less pronounced for Piriform cortex and ventral Pallidum, potentially due to lower signal-to-noise ratios at deep brain regions. Bilateral connectivity appeared less strong in TG compared to WT, a finding consistent with earlier studies in mouse models of amyloidosis^53,73^. Seed-based FC maps were not restricted only to bilateral neuroanatomical regions or networks, but transgressed regional boundaries and displayed connectivity with other brain regions. This finding is consistent with the apparent decreased segregation of brain systems at old ages^74^. Decreased network segregation helps explain the apparent involvement of the motor cortex in the DMN.

The two short QPPs that marked group differences (QPP WT and QPP TG) displayed temporal overlap with each other when the matching counterparts were identified across groups (Fig. 2). Combined with the observation that these short high contrast QPPs displayed a reduced bi-phasic nature, in contrast to prior work in young mice^27^, this may indicate that QPP WT and QPP TG actually pertain to one longer QPP. The latter is common across groups, but its spatiotemporal structure is altered in TG, leading to altered occurrences rates and shapes of short QPPs, i.e. sub-QPPs, in TG. Decreased bi-phasic structure, altered occurrence rates and spatiotemporal shape can explain the differing effects of QPP regression.

Further, in both WT and TG, we observed a similar QPP that displayed overlap with the global signal (QPP GS), and occurred significantly less in TG compared to WT. A similar QPP was previously observed in mice and humans, where it also displayed a temporal coincidence with DMN-TPN anti-correlation QPPs^26,27^. Decreased network segregation, together with decreased network FC and spatial integrity of resting state networks at older ages^75,76^, may explain the sole detection of global signal-like QPPs at longer window sizes, whereas prior work in healthy young mice also determined that longer QPPs, similar to QPP WT, could still be observed when no global signal regression was performed^27^. In supplementary analysis, we obtained QPPs after performing global signal regression (Supplementary Figs S1 and S7). Global signal-like QPPs could no longer be detected, but instead only high contrast QPPs were observed, which occurred considerably less at window sizes longer than 3 s. These observations support that at older ages, long QPPs with DMN-TPN anti-correlation are less reliably detected. Additional analysis confirmed that short QPPs were consistent with one another regardless of performing global signal regression (Supplementary Fig. S7). Altogether, these findings support our choice for analysing the presented data without global signal regression.

Notably, the global signal, i.e. the average intensity across all brain voxels, has often been regarded as a nuisance variable that is removed from the data through linear regression. There is however high controversy to its meaning and to the question if global signal regression should be performed^77^. Recent literature indicated that the global signal contains relevant neuronal information^78^. We therefore performed a supplementary analysis in which we determined the effect of global signal regression on DMN and DMN-TPN FC and used these measures to perform classification (Supplementary Figs S7 and S8). Global signal regression significantly reduced the classification performance of DMN and DMN-TPN (Δ)FC compared to normal DMN FC. Additionally, after global signal regression, QPP GS detection was abolished, while QPP WT and QPP TG were unaffected (Supplementary Fig. S7). This confirmed that the global signal carried important information to dissociate between groups and that QPP GS captured a specific global-signal like dynamic with potential neuronal relevance.

Interestingly, the global signal been linked to changes in arousal, suggested to be modulated through neuromodulation pathways^78–81^. The cholinergic system has been shown to be compromised in both the somatosensory and cingulate cortices of 17 months old TG2576 mice, suggesting a source of altered DMN-TPN dynamics and an interpretation for the lower occurrence rate of QPP GS in TG animals^82^. The global signal has further been indicated to reflect a measure of brain metabolism^83^. A study in 18 months old TG2576 mice showed reduced glucose uptake across the brain, providing another explanation for lowered QPP GS occurrence in TG. Lastly, global signal regression has been shown to reduce FC variability and increase spatial specificity of RSNs because shared variance is taken out of the data^77^. This supports that reduced variance of DMN FC in WT animals after QPP GS regression was expected. Larger variability in WT animals might represent a broader range of basal brain metabolism that contributes to global variance. Notably, observations of diminished FC after QPP WT and QPP TG regression could also be expected given a reduction of shared variance between related brain areas.

In TG animals DMN FC was significantly lower compared to WT. Studies in human patients with Alzheimer’s disease have consistently revealed perturbed DMN FC, and in particular decreased FC with regard to the posterior cingulate cortex^11,68^. The latter is a core DMN area, homologous to the mouse cingulate cortex^51^. Supporting the observations of aberrant network FC, DMN areas display high overlap with the spatial distribution of amyloid plaques in Alzheimer’s disease patients^84,85^. Amyloid itself has been correlated to decreased network FC in clinically healthy subjects with high amyloid burden^86–88^. In the current study, we investigated TG2576 mice, an amyloidosis model in which plaque development starts at the age of 9–11 months and progressively increases across aging^56,57^. At 18 months of age, the cingulate cortex and other DMN areas display high plaque loads^89^. The vulnerability of DMN areas in Alzheimer’s disease has been suggested to be the consequence of high baseline activity earlier in life, leading to increased levels of soluble amyloid and plaque accumulation later on^89–92^. A previous rsfMRI study in TG2576 mice revealed that soluble Aβ, before the onset of plaque deposition, caused hypersynchronous activity in the DMN hippocampus area, while after the onset of plaques, an overall decrease in network FC was observed^54^. Overall, our current findings of aberrant DMN activity, together with the pathological hallmarks of network FC and amyloid in TG2576 mice, match the findings and theoretical frameworks for human Alzheimer’s disease.

Furthermore, two human studies revealed that Alzheimer’s disease caused a disruption of DMN-TPN anti-correlation^17,18^. While in our study we did not observe this change with conventional FC analysis, QPPs did reveal that DMN-TPN dynamic anti-correlation was compromised in TG. The lack of this observation with conventional FC measures in our study might be a consequence of the limited single-slice network investigation or anesthesia. Medetomidine is a vascular constrictor and interacts with α2-adrenorecptors that show their highest expression in cortical areas^93–95^. Resultantly, changes in DMN-TPN FC might be masked. On the other hand, as far as we know, only two studies clearly identified disturbed DMN-TPN FC in humans. The fact that QPPs were still detected, despite sedation, suggests they might represent a more sensitive tool to investigate aberrant DMN-TPN interaction.

A study in APPswe mice used whisker stimulation and voltage sensitive dye imaging to correlate Aβ plaques with abnormal neural population responses in the somatosensory cortex and with reduced cortical synchronization during spontaneous activity^96^. This matches our observation of a dysfunctional TPN. On the other hand, functional MRI studies in 19 months old TG2576 mice and 25 months old APP23 mice revealed a disrupted cerebrovasculature in the somatosensory cortex^97,98^. In line with these studies, it has been shown in TG2576 mice that dense-core amlyloid plaques are mainly centered on vessel walls, contributing to cerebral amyloid angiopathy and microvascular abnormalities^99^. A compromised cerebrovasculature might contribute to altered DMN and TPN BOLD dynamics. However, a recent study in humans showed that only Alzheimer’s patients without cerebrovascular disease showed diminished DMN FC^100^, suggesting a neuronal origin for our findings.

QPPs have been proposed as a time-varying process that contributes to FC in the brain areas it involves^19,32,34,50^. We presented an analytical framework in which QPPs were linearly regressed out of the BOLD image series in order to evaluate how QPPs may reflect FC. While our results suggest a relationship, we cannot claim directionality, that is, QPPs might contribute to FC or FC might shape QPPs. QPPs have previously been directly correlated with infraslow neural activity via simultaneous electrophysiology and MRI recordings^29–31,33,35^. Combined with our current findings, this suggests QPPs may play an active role in contributing to FC. There are however additional limitations for interpreting our findings, given that the BOLD signal displays non-linear properties, which give rise to a combination of linear and non-linear dynamics^101,102^. Linear regression models are commonly used in rsfMRI, but they cannot fully account for non-linear effects. Our classification results support that a linear regression strategy may still be valuable, but future studies will need to establish algorithms and ground truths that can more delicately tease out non-linear contributions of QPPs to FC.

DMN FC displayed high classification performance. This is consistent with clinical classification of Alzheimer’s disease based on DMN FC^15,24,103,104^ and meta-analysis reviews that implicate DMN FC as the most relevant rsfMRI biomarker for Alzheimer’s disease^16,68,105^. DMN-TPN FC displayed low classification performance, which is in congruence with a lack of group differences based on this measure. QPP occurrence rates did not outperform DMN FC classification performance, but some FC-measures obtained after QPP regression significantly increased performance. After regression of QPP GS, DMN FC allowed 100% classification, indicating that this QPP contributed to DMN FC variability across subjects. This was particularly the result of decreased DMN FC standard error in WT mice. Regression of QPP WT and QPP TG did not significantly increase DMN or DMN-TPN FC classification performance, but the consequent subject-wise DMN and DMN-TPN ΔFC allowed 98–100% classification. This indicates that QPP WT and QPP TG played an important role in shaping DMN and DMN-TPN FC group differences. The combined model also allowed 100% classification and indicated that DMN and DMN-TPN ΔFC after QPP TG regression were most important for classification. This finding is important when considering potential translation to clinical practice, suggesting that patient-specific QPPs should be evaluated with regard to a healthy population, rather than vice versa. The combined model further indicated that DMN-TPN ΔFC after QPP WT and QPP TG regression were also highly important for classification. It has been suggested that DMN-TPN FC might help improve Alzheimer’s disease classification^13^. Our results contribute to this notion and highlight the importance of QPPs in enabling this improvement. While QPP-derived FC measures significantly increased performance compared to normal FC measures, the resultant 100% classification may in part be explained by the pathological severity of the TG2576 model at old age.

To validate that QPPs could truly provide novel insights and optimize classification, we performed two supplementary analyses. We used an additional selection criterion to isolate different QPPs between WT and TG, identifying those QPPs that showed the highest ratio of occurrence between their STC and pSTC with the target group. This strategy provided nearly identical QPPs as described in the manuscript, which had the same effect on FC after QPP regression (Supplementary Fig. S9). We also determined resting state networks across groups, using ICA, and regressed components with similar features as QPP WT. While there was a similar trend to diminish DMN FC, DMN-TPN FC was not altered and classification was not improved (Supplementary Fig. S10). QPPs thus provided superior classification compared to conventional rsfMRI approaches.

In the current study, all analyses were restricted to a single brain slice. While the DMN and TPN are represented in the respective slice, they do describe larger networks that could not fully be investigated. For instance, the connection between cingulate cortex and hippocampus, commonly affected in Alzheimer’s disease patients^11^, could not be observed. Future rodent studies with larger brain coverage, but lower spatial resolution, might explore the full extent of compromised DMN FC and altered QPP dynamics. In human studies, these restrictions do not apply, given that QPP temporal lengths are approximately 20 s and can be sufficiently sampled with conventional acquisition schemes^26^. This simplifies the translation of QPP research to clinical studies. QPP occurrence rates and sliding correlation values also appeared higher in humans than in mice^25–27^, which is promising for robust single subject QPP detection in commonly short clinical rsfMRI acquisitions. In the current study, QPPs were determined based on the concatenated images of single subjects, similar as to what is done in a group ICA. This provides a strategy that increases the reliability of derived QPPs and still allows their evaluation at the single subject level. Prior work in mice and humans additionally suggests that single subject QPPs are very similar to group level QPPs. This is promising to envision single subject analysis strategies that can enable classification. While in the current study the heuristic was used to run the spatiotemporal pattern finding algorithm 500 times per window size, which subsamples the data, it is currently unclear what number of QPPs would have to be determined in humans to enable detection of relevant group differences. This may be established in future studies. Further, in the current study we focussed on a mouse model of amyloidosis and obtained findings well in line with clinical rsfMRI research of amyloid pathology. While Aβ is one central hallmark of Alzheimer’s disease, so are tau tangles and neurodegeneration^1^. Future studies in rodent models that co-develop these pathologies will shed further light on the translational value of QPPs. Additionally, we only investigated female mice, which limits the interpretation of our findings with regard to patient studies. Finally, while our results provide novel insights into aberrant neural dynamics, multi-modal imaging studies, combining haemodynamic and neural readouts, will be required to shed light on how exactly the neural and/or vascular functionality of the DMN and TPN are compromised in Alzheimer’s disease.

## Conclusion

In this study, we applied drsfMRI for the first time to an aged mouse model of Alzheimer’s disease and revealed that quasi-periodic patterns of BOLD activity reflect aberrant network FC of two major resting state networks, the DMN and TPN. Additionally, FC measures obtained through quasi-periodic patterns allowed full subject classification, outperforming conventional FC-measures. These findings are of high translational value and provide a novel analysis strategy to advance rsfMRI research of neurological disorders.

## Electronic supplementary material

Supplementary Methods and Figures
