# Author Correction: Quasi-Periodic Patterns of Neural Activity improve Classification of Alzheimer’s Disease in Mice

**DOI:** 10.1038/s41598-019-53432-7

**Published:** 2019-11-08

**Authors:** Michaël E. Belloy, Disha Shah, Anzar Abbas, Amrit Kashyap, Steffen Roßner, Annemie Van der Linden, Shella D. Keilholz, Georgios A. Keliris, Marleen Verhoye

**Affiliations:** 1 Department of Pharmaceutical, Veterinary and Biomedical Sciences, Bio-Imaging Lab, University of Antwerp, Universiteitsplein 1, 2610, Wilrijk, Antwerp, Belgium; 2 Department of Biomedical Engineering, Emory University, 1760 Haygood Dr. NE, Atlanta, GA 30322 USA; 3 Department of Neuroscience, Emory University, 1760 Haygood Dr. NE, Atlanta, GA 30322 USA; 4 Department of Biomedical Engineering, Emory University and Georgia Institute of Technology, 1760 Haygood Dr. NE, Atlanta, GA 30322 USA; 5 Paul Flechsig Institute for Brain Research, University of Leipzig, Liebigstraße 19. Haus C, 04103 Leipzig, Germany

Correction to: *Scientific Reports* 10.1038/s41598-018-28237-9, published online 03 July 2018

The Author Contributions section in this Article is incorrect.

“M.E.B. wrote manuscript, designed research, performed research, performed analysis, and designed analysis tools. D.S. contributed valuable comments to research design and data analysis. A.A., A.K., and S.R. contributed material and analysis tools. A.V.d.l., S.D.K., G.A.K. and M.V. designed research and provided input on data analysis.”

should read:

“M.E.B. wrote manuscript, designed research, performed research, performed analysis, and designed analysis tools. D.S. contributed valuable comments to research design and data analysis. A.A., A.K., and S.R. contributed material and analysis tools. A.V.d.l., S.D.K., G.A.K. and M.V. designed research and provided input on data analysis. G.A.K. and M.V. contributed equally to this work.”

