# Supplementary material for: Quasi-Periodic Patterns of Neural Activity improve Classification of Alzheimer’s Disease in Mice
Source: Sci Rep. 2018 Jul 3;8:10024. doi: 10.1038/s41598-018-28237-9 (PMC6030071; doi:10.1038/s41598-018-28237-9)
Supplement: Supplementary file 1 — Supplementary Methods and Figures [file 41598_2018_28237_MOESM1_ESM.pdf]

Quasi-Periodic Patterns of Neural Activity improve Classification of Alzheimer's Disease in Mice

Authors

Michaël E. Belloy[1-2], Disha Shah[1], Anzar Abbas[3], Amrit Kashyap[4], Steffen Roßner[5], Annemie Van der Linden[1], Shella D. Keilholz[2-4], Georgios A. Keliris[1], Marleen Verhoye[1].

Supplementary Material

Supplementary Methods

Animals – MRI handling procedures

During MRI handling procedures, animals were anesthetized with 2 % isoflurane. Animals were positioned in the magnet by securing their heads with ear bars and fixing incisors over a bite bar. Ophthalmic ointment was applied to the eyes and a rectal temperature probe was used to monitor animal body core temperature. Body temperature was kept stable at 37 °C via a hot air supply (MR-compatible Small Animal Heating System, SA Instruments, Inc.). A pressure sensitive pad was used to monitor breathing rate and a fiber-optic pulse oximeter, positioned over the tail, to asses heart rate and O_2 saturation (MR-compatible Small Animal Monitoring and Gating system, SA Instruments, Inc.).

Animals then received a 0.3 mg/kg bolus injection of medetomidine (Domitor, Pfizer, Karlsruhe, Germany), after which isoflurane was lowered to 0.4 %. Starting at 15 min post-bolus, a subcutaneous catheter allowed continuous infusion of 0.6 mg/kg/h medetomidine. Functional resting state scans were acquired 30 min post-bolus, lasting 20 min. Great care was taken to keep procedures and conditions identical across animals, with preparatory handling never exceeding 10 min. The choice for a relatively higher anesthesia regimen was based on prior knowledge at our lab, and observations made during pilot tests, that animals would not remain sedated with low anesthesia levels. These observations can be explained given the known differences in neuronal activity and behavior across different mouse strains, and given the increased anxiety levels in multiple mouse models of Alzheimer's disease[1–3].

Identifying quasi-periodic pattern temporal lengths – low contrast QPPs

We used a data-driven processing tool that allows one to determine the most representative window length for a specific type of spatiotemporal pattern. We term this strategy fractional average correlation (FA) and used it to identify the window length of the global-signal like low-contrast QPPs. This was done independently in both WT and TG animals, indicating for each group an ideal window length at 6 s (**Supplementary Fig. S2**).

A set of global-signal like low-contrast QPPs was chosen so that each QPP in the set was of a different window length, ranging from 4.5 s to 15 s (at 3 s no global-signal like QPPs were

clearly observed). A total of eight QPPs was thus chosen. Each individual QPP in this set was then subdivided into all possible consecutive fractions of a fixed length, specified by the smallest window size investigated. In our analysis the smallest window size was 6 TRs (3 s), meaning that a QPP of e.g. 18 TRs was divided into 13 fractions of 6 consecutive images, each of them shifted by 1 TR (0.5 s). Each of the individual fractions from a QPP at a respective window size were then treated as a reference, and the maximal cross-correlation was calculated with respect to a non-fractioned 'target' QPP at another window size. The average of resultant cross-correlation values from this comparison indicates how many fractions the reference and target QPP had in common, i.e. the FA. FA thus allows one to determine a measure of QPP similarity, which is independent of their spatiotemporal length. The FA-value was then calculated for all combinations of window sizes. All FA-values were represented in an n x n matrix, where n indicates the number of QPPs and each column indicates the FA-values for a respective reference QPP with each respective (target) QPP in the set. The FA-matrix was finally averaged across its columns to obtain the overall FA for each QPP in the set.

This approach is particularly useful because target QPPs at smaller window sizes than the reference QPP under investigation intrinsically have less fractions in common, given that they only represent a subpart of the larger QPP. Comparing larger reference QPPs with non-matching subparts in short target QPPs forces a decrease in the FA-value. In contrast, long target QPPs likely contain the full spatiotemporal pattern present in the reference QPP and will therefore cause a high FA. When FA-values are averaged across columns (i.e. all reference QPP FA-values with a respective target QPP), this will lead to low average FA-values for QPPs shorter than then ideal window length and to high average FA-values for QPPs longer than the ideal window length. The tipping point of increasing FA, before a plateau is reached, reflects the optimal window size (**Supplementary Fig. S2**). A gradual decrease in the plateau is the result of lower QPP integrity at longer window sizes, given that these QPPs displayed lower occurrence rates and therefore less image frames were averaged in order to establish the QPP.

Classification analysis – Additional details

A total of 17 rsfMRI measures were independently used as predictors in elastic net logistic regression models, to classify mice as either WT or TG. Additionally, all 17 measures were used

simultaneously in a combined classification model. We employed the machine learning toolbox available in MATLAB (2017b), making use of the functions 'lassoglm', 'glmval', and 'perfcurve', in order to construct and evaluate the models. The analysis methodology is strongly in line with the one presented in de Vos et al. (2017)[4].

Elastic net regression is a regularization technique for the estimation of generalized linear models. It operates by combining LASSO regularisation (L1) and ridge regression (L2) penalty terms. These penalty terms constrain the size of estimated predictor coefficients, which can be reduced to zero [5]. This effectively allows only the most relevant predictors to be part of the regression model. Thus, for each model, two hyper parameters need to be set: λ, which determines the two penalty sizes, and α, which provides the relative weight of both penalty terms and therefore determines the balance towards either LASSO or ridge optimization.

In elastic net regression, two risks are represented by: 1) over-fitting models with too many predictors, and 2) overestimating classification accuracy by selecting only those hyper parameters that produce the best results. To avoid these biases, we employed a nested 10-fold cross-validation approach [6]. The inner loop was used to determine the hyper parameters with a grid search approach, while the outer loop was used to evaluate the resultant regression models. More specifically, this means that in the outer loop, 90% of the data was used as a training set and 10% as a validation set, which was repeated 10 times to ensure that each subject was part of the validation set once. Each training set of the outer loop was then respectively used to perform a grid search on a set of α values and λ values. For each combination of hyper parameters tested on the inner loop, a 10-fold cross-validation was performed to determine the optimal values, which were used to construct the regression model that was evaluated in the outer loop.

To evaluate classification performance of the resultant models, we constructed receiver operating characteristic (ROC) curves and calculated the area under the curve (AUC). The entire cross-validation procedure described above was repeated 10 times for each model, in order to increase the reliability of the cross-validation error and to determine the mean AUC values[6]. To statistically compare between AUC-values of the investigated models, AUC-values were bootstrapped 2000 times, with stratification, for each pair[7]. Models were compared with two-sided tests. P-values are shown both uncorrected and after Bonferroni correction.

Finally, to investigate the role of global signal regression on classification performance, 4 additional rsfMRI measures, derived after global signal regression, were independently used to construct net logistic regression models. An additional combined model was constructed, where all 21 rsfMRI measures were used simultaneously. Post-hoc Bonferroni correction was adapted to include the additional AUC comparisons.

Independent component analysis (ICA) and regression

Group ICA was performed on WT and TG groups separately (**Supplementary Fig. S3**), and on both groups simultaneously (**Supplementary Fig. S10**), using the GIFT toolbox (v4.0b)[8]. This allowed us to determine RSNs for the respective analyses. For the separate WT and TG analyses, the number of independent components was set to five for both groups. If a higher number was used this led to splitting of bilateral components in the WT group. The presented RSNs of the TG group (**Supplementary Fig. S3**) also appeared split when three or four components were used, indicating that the observation of unilateral components in TG was not solely the result of running the analysis with five components. For the combined group analysis, the number of components was set to 4, based on the same heuristics as described above. The ICA was run on variance-normalized data, filtered between 0.01-0.2Hz, using the Infomax algorithm with no auto-filling of data reduction values. Stability analysis was performed using the ICASSO algorithm, rerunning the ICA 15 times with a minimal cluster size of 5 and maximal of 10. Using the built-in GIFT functionality, SPM12 (Statistical parametric mapping) was used to obtain contrast images per subject for all components. The latter were used in one sample T-tests for all components to obtain significant group level RSNs ($p < 0.05$, FDR corrected, threshold 4 voxels).

To evaluate the contribution of RSNs to FC in both groups (**Supplementary Fig. S10**), the GIFT toolbox was used to regress group-level spatial maps (RSNs) into individual subject level data. This establishes RSN time courses at the subject level and can be used to obtain subject specific spatial maps that represent the group-level RSNs. The final time courses of the RSNs were used for further regression analysis. For all components, we determined the percentage of variance explained by regressing that component out of the data, i.e. percentage of variance = 100 x ($1-\frac{Residual\ variance}{Total\ variance}$). The two components that indicated the highest percentage of variance

explained were then regressed in order to evaluate their effect on DMN- and DMN-TPN-like FC, and classification.

References

1. Crawley, J. N. *et al.* Behavioral phenotypes of inbred mouse strains: implications and recommendations for molecular studies. *Psychopharmacology (Berl).* **132,** 107–124 (1997).
2. Webster, S. J., Bachstetter, A. D., Nelson, P. T., Schmitt, F. A. & Van Eldik, L. J. Using mice to model Alzheimer's dementia: an overview of the clinical disease and the preclinical behavioral changes in 10 mouse models. *Front. Genet.* **5,** 1–23 (2014).
3. Shah, D. *et al.* Early pathologic amyloid induces hypersynchrony of BOLD resting-state networks in transgenic mice and provides an early therapeutic window before amyloid plaque deposition. *Alzheimer's Dement.* **12,** 964–976 (2016).
4. De Vos, F. *et al.* A comprehensive analysis of resting state fMRI measures to classify individual patients with Alzheimer's disease. *Neuroimage* **167,** 62–72 (2017).
5. Friedman, J., Hastie, T. & Tibshirani, R. Regularization Paths for Generalized Linear Models via Coordinate Descent. *J. Stat. Softw.* **33,** 1–22 (2010).
6. Krstajic, D., Buturovic, L. J., Leahy, D. E. & Thomas, S. Cross-validation pitfalls when selecting and assessing regression and classification models. *J. Cheminform.* **6,** 10 (2014).
7. Hanley, J. A. & Mcneil, B. J. A method of Comparing the Areas under Receiver Operating Characteristic Curves from the Same Cases. *Radiology* **148,** 839–843 (1983).
8. Calhoun, V., Pearlson, G. & Adali, T. Independent Component Analysis Applied to fMRI Data : A Generative Model for Validating Results. *J. VLSI Signal Process.* **37,** 281–291 (2004).

Supplementary Figures

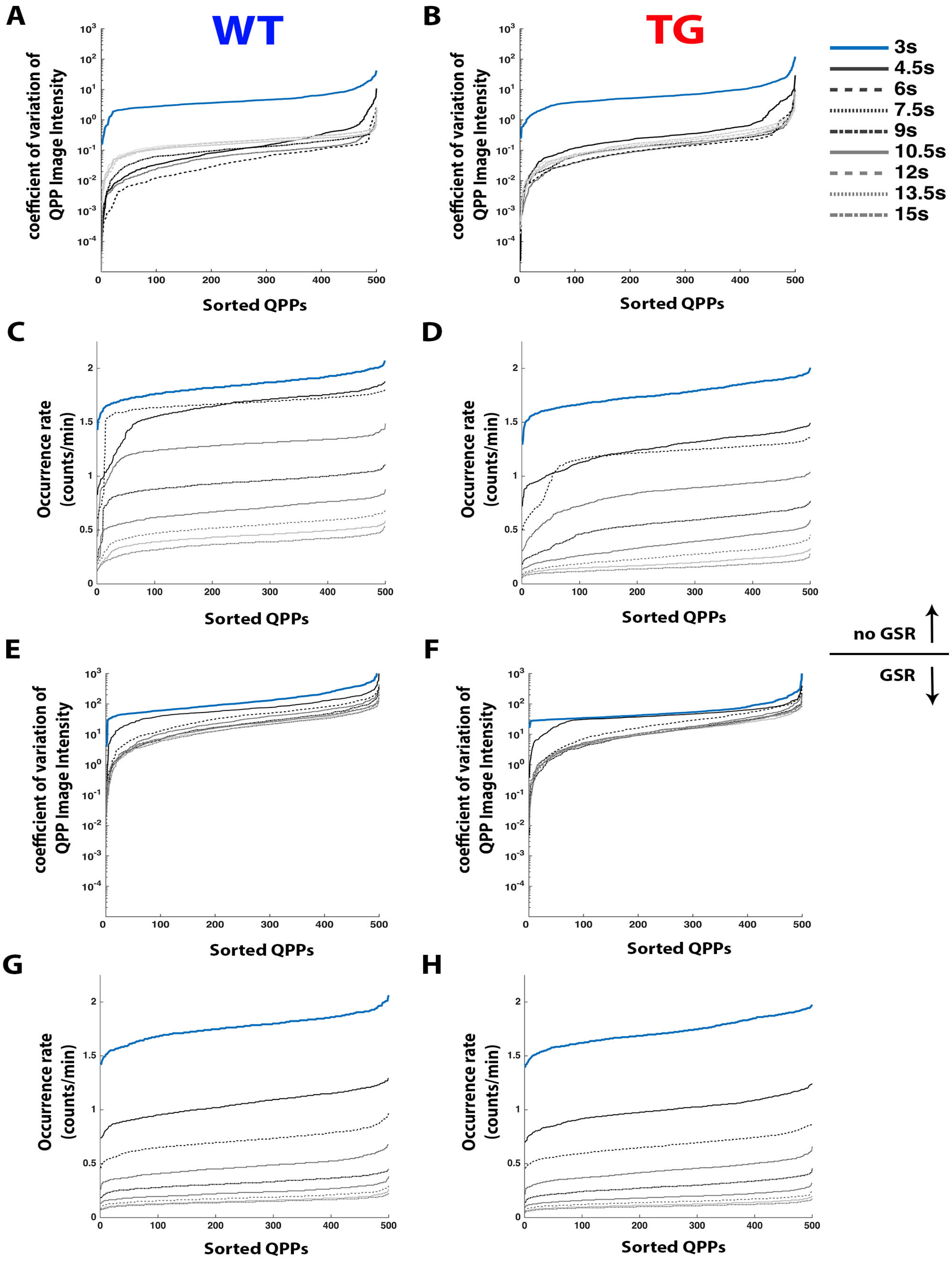

I

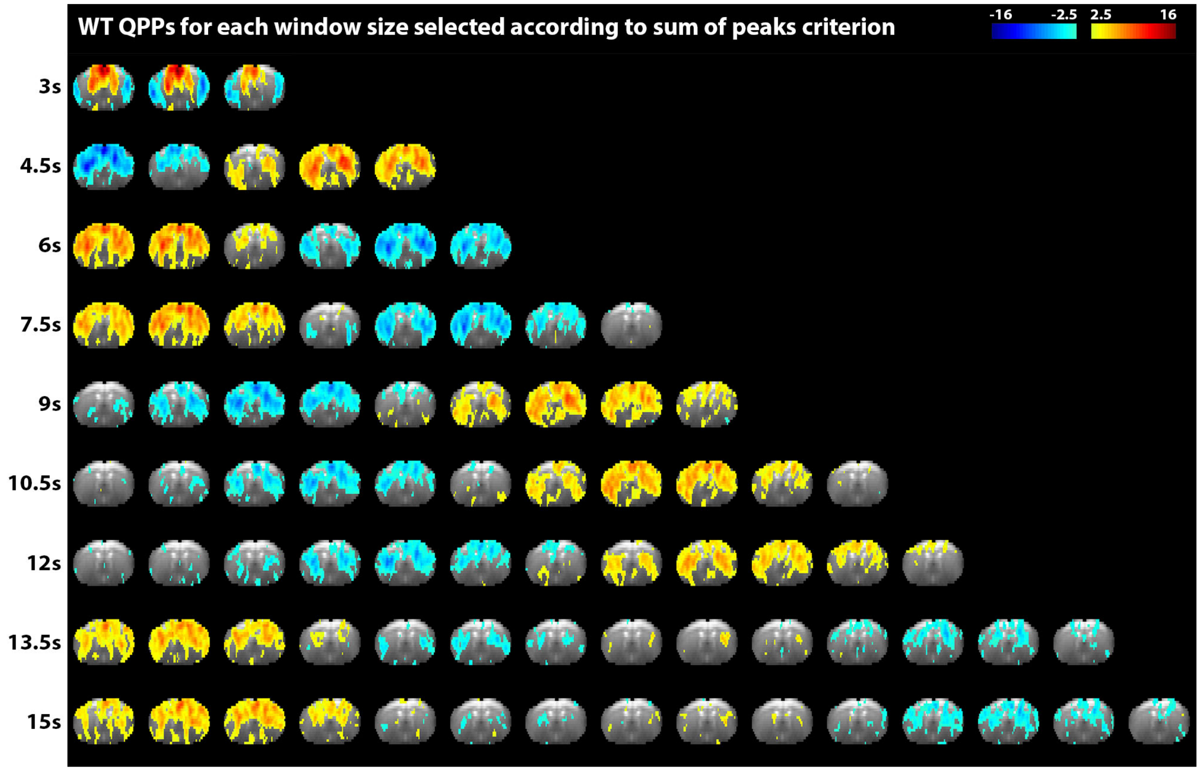
WT QPPs for each window size selected according to sum of peaks criterion
-16
-2.5
2.5
16
3s
4.5s
6s
7.5s
9s
10.5s
12s
13.5s
15s

J

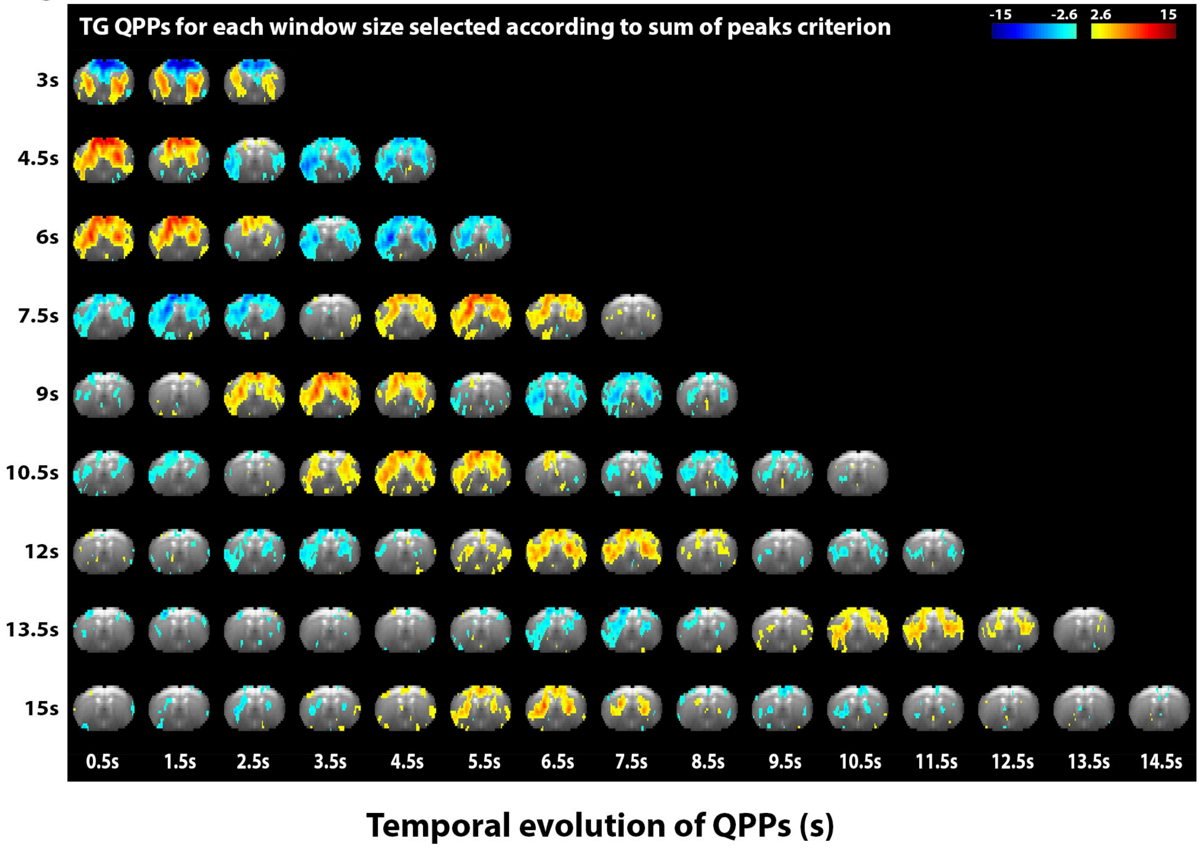
TG QPPs for each window size selected according to sum of peaks criterion
-15
-2.6
2.6
15
3s
4.5s
6s
7.5s
9s
10.5s
12s
13.5s
15s
0.5s
1.5s
2.5s
3.5s
4.5s
5.5s
6.5s
7.5s
8.5s
9.5s
10.5s
11.5s
12.5s
13.5s
14.5s
Temporal evolution of QPPs (s)

Supplementary Figure 1. QPP occurrence rates and contrast

A-D) Analysis without GSR, and **E-H)** with GSR, split into **A,C,E,G)** WT group, and **B,D,F,H)** TG group. **A,B,E,F)** For each investigated window length, all 500 WT QPPs were displayed by their mean coefficient of variation (C_V) of image intensities across time: $\overline{\mathrm{QPP}Cv}$ (cfr. material & methods). At 3 s window lengths without GSR, QPPs displayed clear image contrast, causing high $\overline{\mathrm{QPP}Cv}$ values. At longer window lengths without GSR, QPPs globally involved several brain areas simultaneously, leading to a loss of contrast and low $\overline{\mathrm{QPP}Cv}$ values. After GSR, all QPPs displayed clear image contrast, causing high $\overline{\mathrm{QPP}Cv}$ values. **C,D,G,H)** Subject mean occurrence rates across the respective groups for all 500 QPPs at each window size. Note that global signal-like QPPs occurred more frequently in WT compared to TG (C-D). Note that after GSR, occurrence rates of QPPs longer than 3 s dropped steeply. **A-H)** To aid visual clarity, panels display QPPs sorted according to the described parameter. **I-J)** Example of QPPs obtained at each window size without GSR, for WT (I) and TG (J). The presented QPPs were determined based on the selection criterion of Yousefi et al. (2018). QPPs are displayed as thresholded T-maps, overlain on respective brain images (one-sample T-test, FDR $p<0.05$) ***Abbreviations***. *GSR, global signal regression; DMN, Default Mode network; TPN, Task-Positive network.*

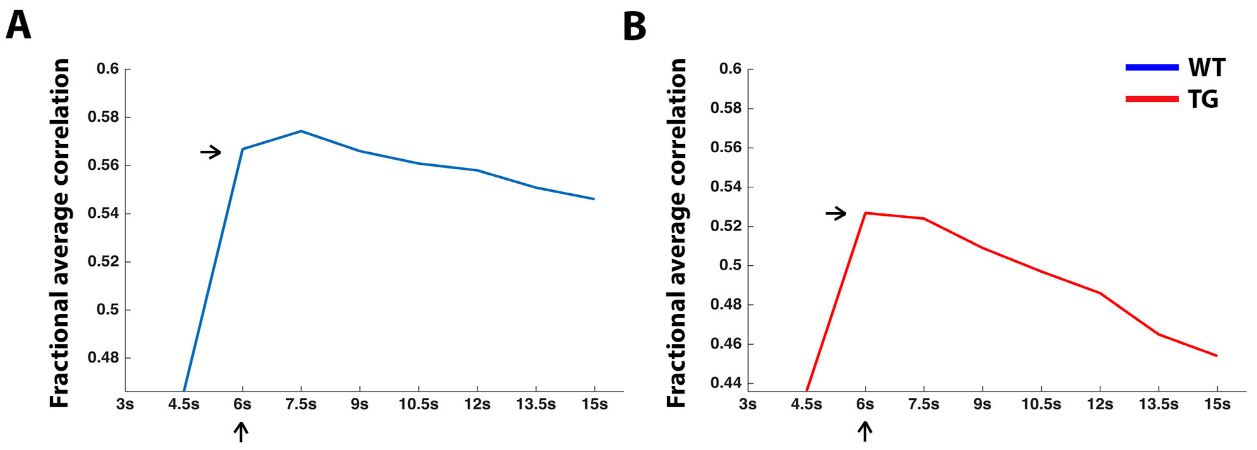

Supplementary Figure 2. The window length of the global-signal like Quasi-Periodic pattern

Visual inspection, and findings presented in Supplementary Fig. 1, indicated that longer global-signal like QPPs were consistent between WT and TG. Essentially, at all window lengths longer than 3 s, mostly the same global-signal like QPP was observed, regardless of the investigated temporal length. Therefore, using a data-driven analysis method, fractional average correlation (cfr. Supplementary Methods), we determined the true window length of the global-signal like QPP to be 6 s in both **A)** WT and **B)** TG groups. In brief, fractional average correlation measures the amount of overlap between spatiotemporal patterns of different lengths. Short patterns, which are below the true length of the spatiotemporal dynamic under investigation, only display partial overlap with longer ones. Once pattern lengths exceed the true length, their overlap peaks and eventually declines because of lower QPP spatial integrity at longer window sizes. The tipping point of the curve that describes this value determines the true QPP length.

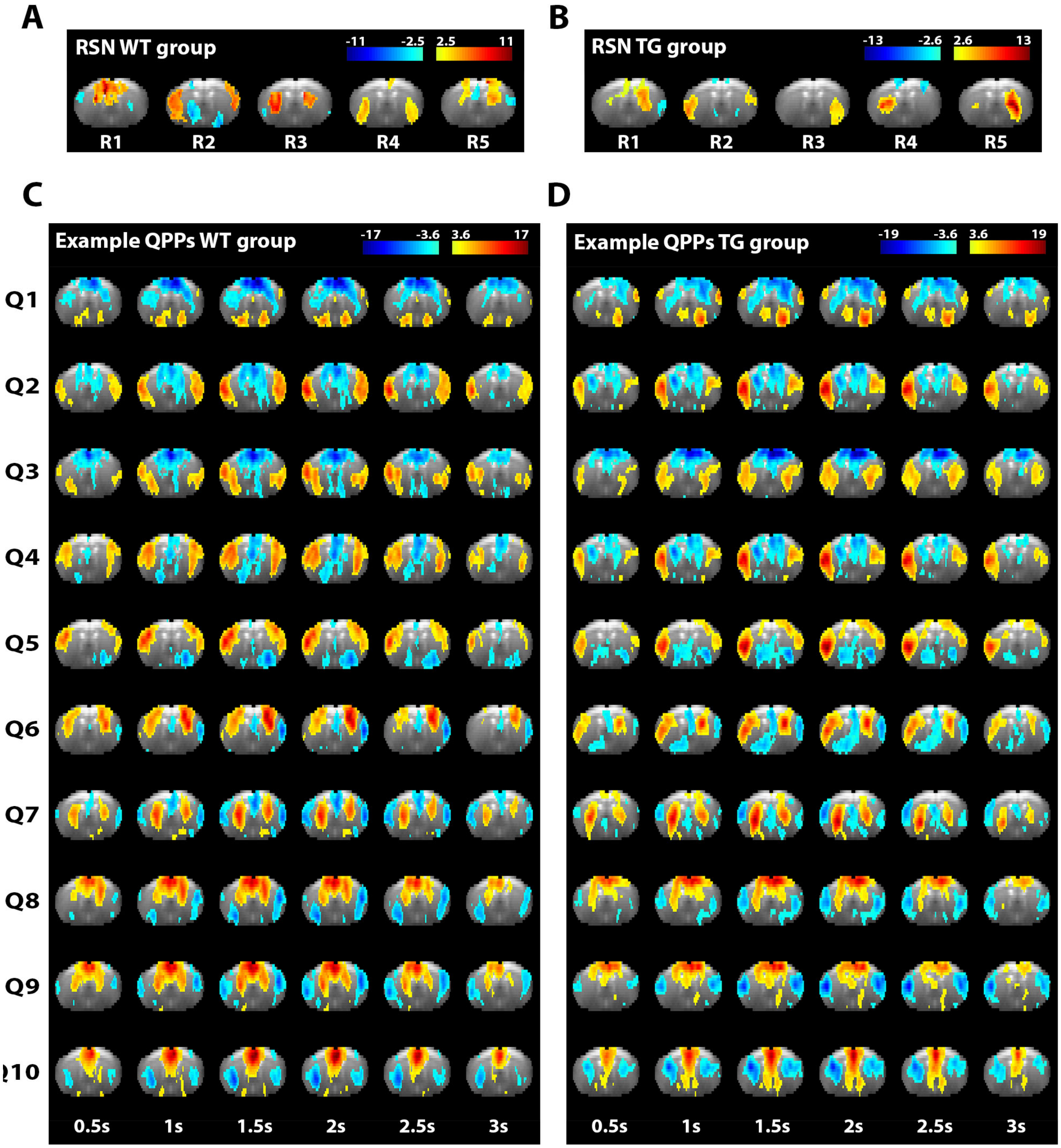

Supplementary Figure 3. Illustration of short quasi-periodic patterns and their spatial correspondence to ICA-derived resting state networks.

A-B) ICA-derived RSNs for WT (A) and TG groups (B). RSNs are displayed as thresholded T-maps, overlain on respective brain images (one-sample T-test, FDR p<0.05). **C)** Illustration of 10 visually identified 3 s QPPs in the WT group. QPPs are displayed as thresholded T-maps,

overlain on respective brain images (one-sample T-test, FDR $p<0.001$). Note the richness of different spatiotemporal patterns with bilateral structure that match with neuroanatomical areas. Further, note the observation of QPPs in different phases (inverted activity profiles): e.g. Q2-Q3 versus Q8-Q9. **D)** Same QPPs as shown in (C), determined in the TG group via pSTC matching procedure (cfr. Fig. 1). QPPs are displayed as thresholded T-maps, overlain on respective brain images (one-sample T-test, FDR $p<0.001$).

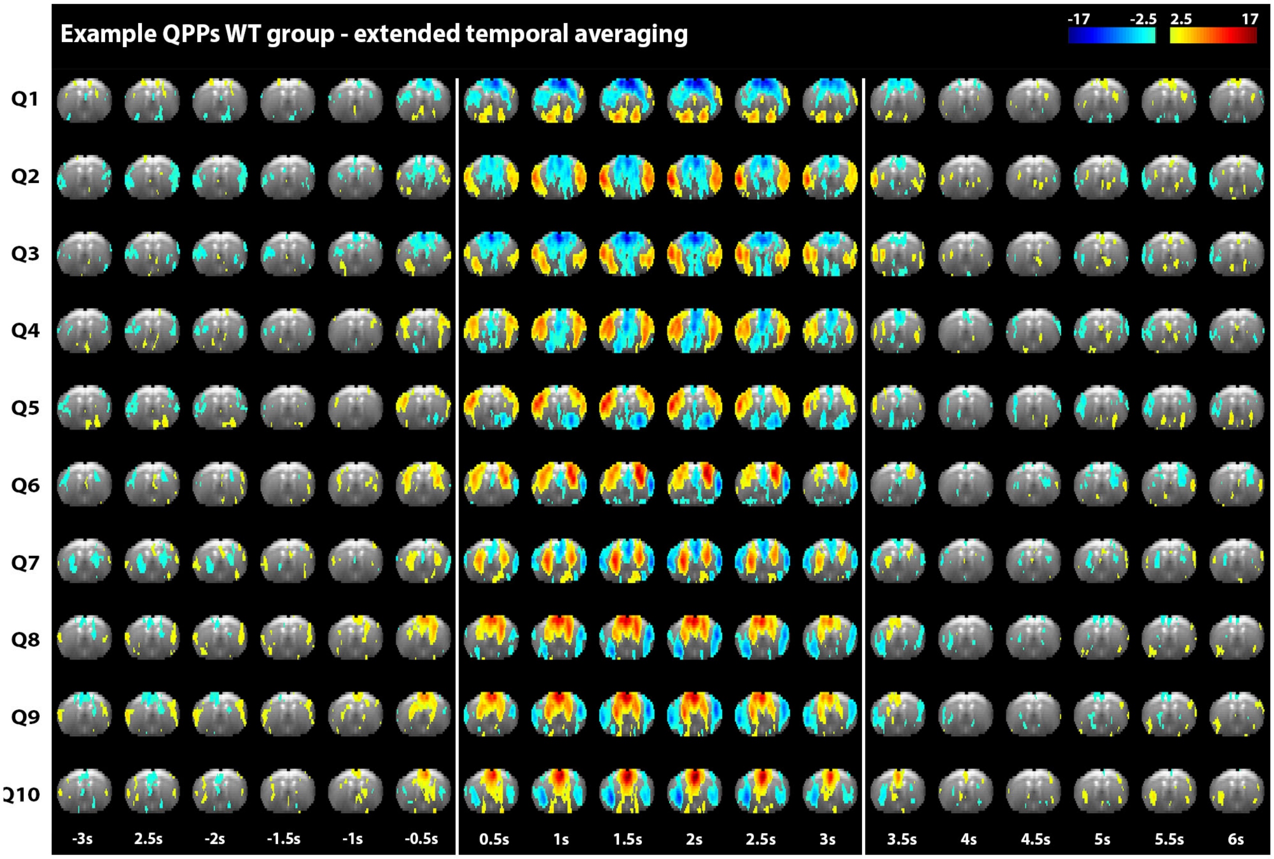

Supplementary Figure 4. Illustration of short quasi-periodic patterns and their bi-phasic nature

C) Illustration of 10 visually identified 3 s long QPPs in the WT group. These QPPs are the same as presented in Supplementary Fig. S3. Additionally, image frames 3 s before and 3 s after the window length of the QPP were averaged. The relevant time points for image selection are still based on the BOLD data within the 3 s window length used for the spatiotemporal pattern finding algorithm. This figure therefore provides a temporal extension of the short QPPs. QPPs are displayed as thresholded T-maps, overlain on respective brain images (one-sample T-test, FDR $p<0.05$). Note that biphasic structure may be observed for several QPPs, but less reliably compared to the center 3 s sections. This figure supports the observation that short high contrast QPPs are part of longer QPPs that are less reliably observed in the current study.

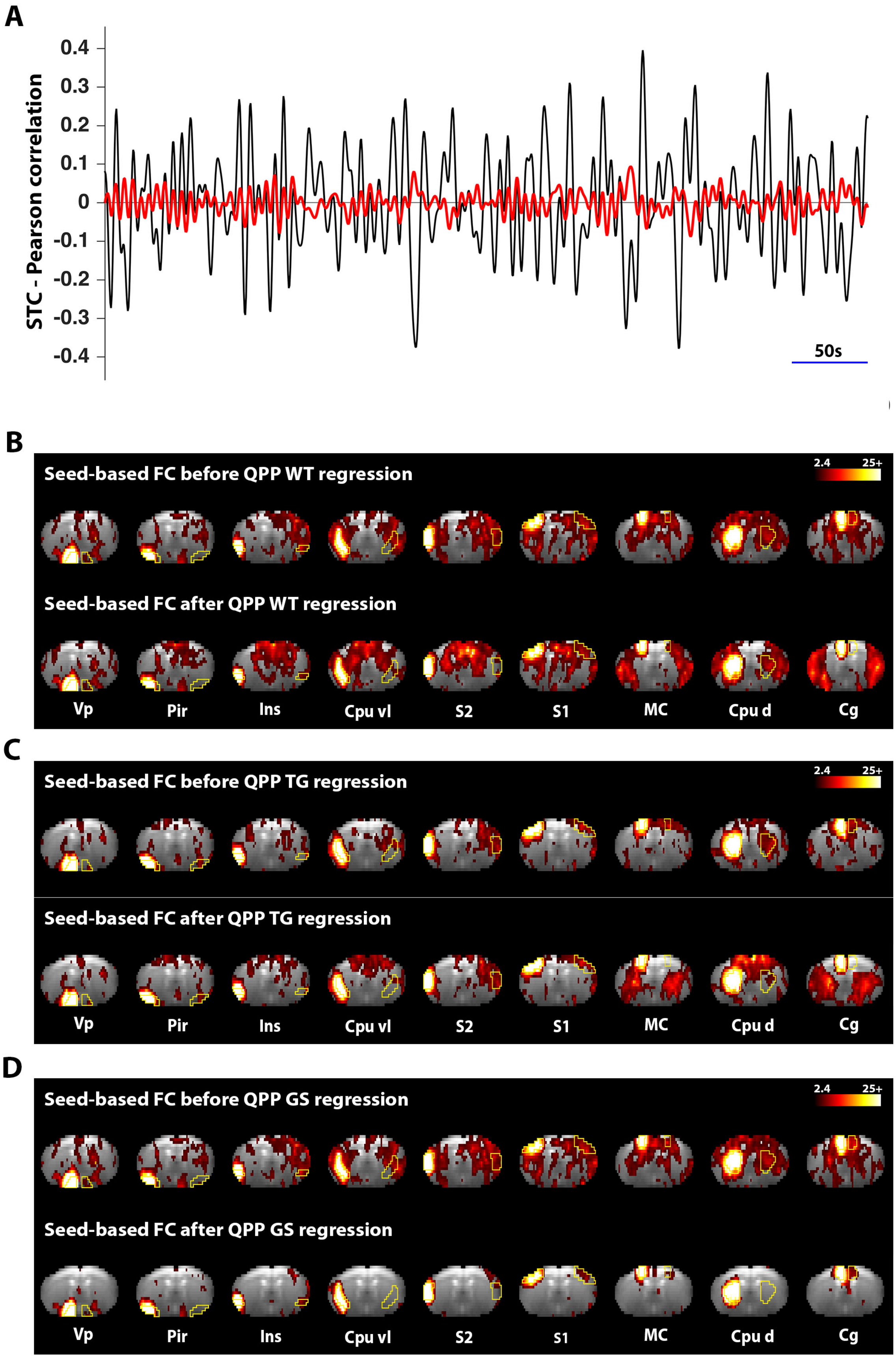
A
STC - Pearson correlation
0.4
0.3
0.2
0.1
0
-0.1
-0.2
-0.3
-0.4
50s
B
Seed-based FC before QPP WT regression
2.4
25+
Seed-based FC after QPP WT regression
Vp
Pir
Ins
Cpu vl
S2
S1
MC
Cpu d
Cg
C
Seed-based FC before QPP TG regression
2.4
25+
Seed-based FC after QPP TG regression
Vp
Pir
Ins
Cpu vl
S2
S1
MC
Cpu d
Cg
D
Seed-based FC before QPP GS regression
2.4
25+
Seed-based FC after QPP GS regression
Vp
Pir
Ins
Cpu vl
S2
S1
MC
Cpu d
Cg

Supplementary Figure 5. Linear regression of Quasi-periodic patterns: evaluation of regression performance and functional connectivity before and after regression

This figure complements Fig. 2 & Fig. 3 in the main manuscript, where QPPs and the FC differences due to QPP regression are described. **A)** In black is displayed a 500 s excerpt of an STC in one subject of the WT group. This STC was obtained by sliding window correlation of QPP WT with the respective image series. In red the same QPP WT is compared with the respective image series after it has been linearly regressed out of the image series. Note the overall clear decrease in magnitude of correlations. **B-D)** Panels illustrate FC before and after regression of: **B)** QPP WT in WT image series, **C)** QPP TG in TG image series, and **D)** QPP GS in WT image series. Each panel displays seed-based FC maps for left seed regions, which are specified at the bottom of the panel. Seed regions, and their contralateral counterpart, are indicated with yellow contours. Top row indicates FC before QPP regression, and bottom row after. Maps show T-values, based on voxel-wise zFC distributions of all animals in the group from which the respective QPPs were derived. Maps are liberally thresholded to show the full extent of FC (one-sample T-test, $p<0.05$). (A) Top row. Note the overall bilateral FC and also the FC between DMN-like brain areas (Cpu d and Cg). (A) Bottom row. Note the loss of bilateral FC in brain regions that were co-active within the QPP, and the increase in FC between areas that were anti-correlated within the QPP **(cfr. Fig. 2)**. (B-C). Note the similar results as in (A). (B) Top row. Bilateral FC was also observed in TG animals, appearing less prominent compared to WT. ***Abbreviations***. *DMN, Default Mode like network; TPN, Task-Positive like network; Cg, Cingulate area; Cpu d, Caudate Putamen dorsal; MC, motor cortex; S1, somatosensory area 1; S2, somatosensory area 2, Ins, Insular; Cpu vl, Caudate Putamen ventro-lateral; Pir, piriform cortex; VP, ventral pallidum.*

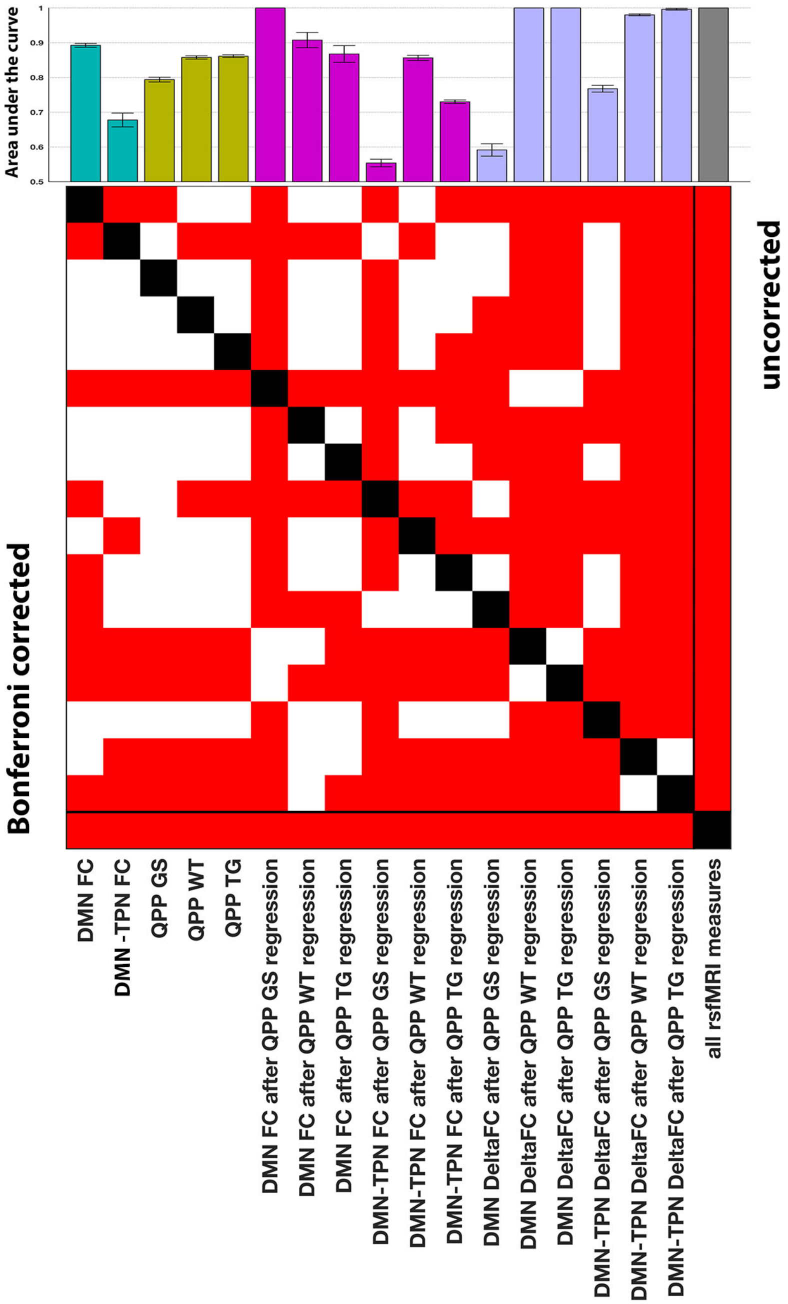

Supplementary Figure 6. Statistical comparison of classification results

Upper panel displays the same mean AUC-values for all rsfMRI measures, as were shown in Fig. 6. Statistical comparisons between the AUC-values are indicated in the matrix below. Red colour indicates $p<0.05$. The upper right side indicates uncorrected p-values, while the lower left bottom indicates p-values after Bonferroni post-hoc correction.

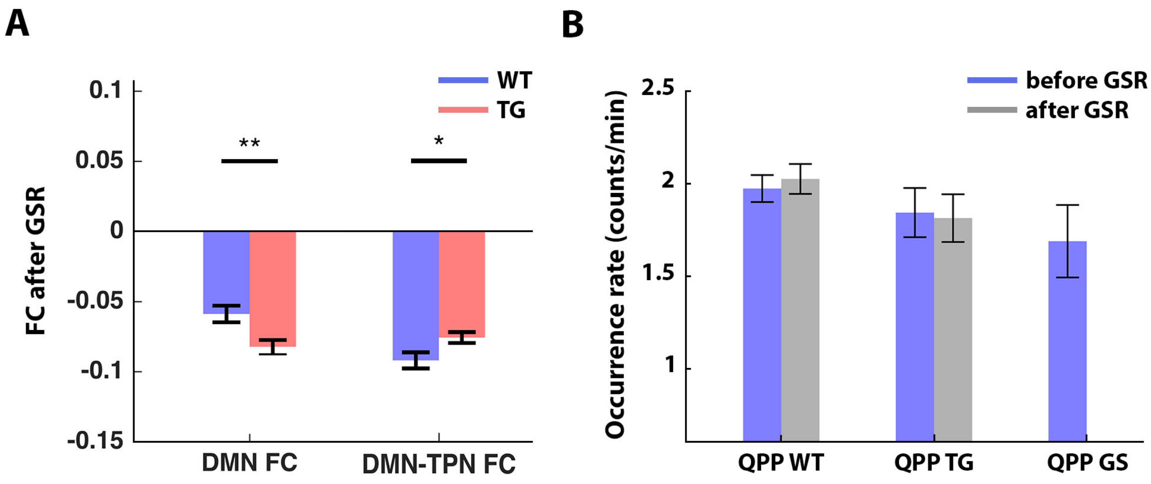

Supplementary Figure 7. The effect of global signal regression on network functional connectivity and quasi-periodic pattern occurrences

A) Subject average zFC-values between (left) all DMN-like area pairs and (right) all DMN-TPN-like area pairs, determined after GSR. Bar graphs display group average FC (two-sample T-test, *p<0.05, **p<0.01, error flags indicate standard error). **B)** Subject occurrence rates for all three investigated QPPs, presented in the main manuscript. Bar graphs display group mean occurrence rates (error flags show standard error). Blue indicates the QPP occurrence rates in the group from which they were originally derived. Using pSTC, the QPPs were compared with the same group's respective image series, after GSR. The resultant occurrence rates are indicated in grey. Note the total loss of occurrences for QPP GS, and the non-significant change for QPP WT and QPP TG (two-sample T-test, p<0.05, error flags indicate standard error). In a separate analysis, QPPs were also directly obtained after performing GSR. Using pSTC, QPP WT and QPP TG could be used to determine matching QPPs after GSR that were highly similar in spatiotemporal configuration (respectively 0.97 and 0.95 spatial cross-correlation values). This validates that 3 s QPPs were the same regardless of performing GSR and supports the presented analysis in the main body of the article. ***Abbreviations***. *GSR, Global Signal Regression; pSTC, project Sliding Template Correlation.*

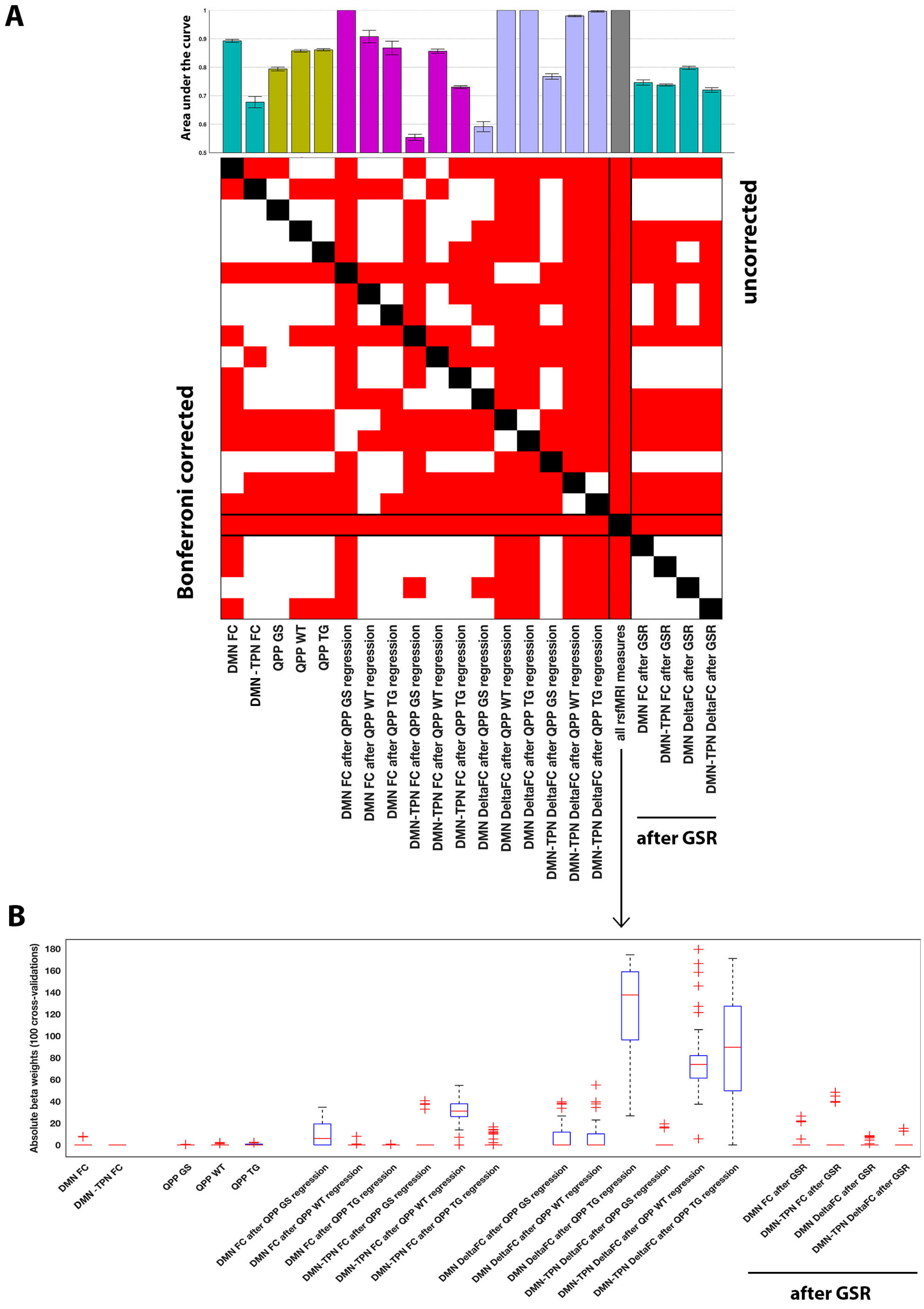

A
Area under the curve
1
0.9
0.8
0.7
0.6
0.5
uncorrected
Bonferroni corrected
DMN FC
DMN -TPN FC
QPP GS
QPP WT
QPP TG
DMN FC after QPP GS regression
DMN FC after QPP WT regression
DMN FC after QPP TG regression
DMN-TPN FC after QPP GS regression
DMN-TPN FC after QPP WT regression
DMN-TPN FC after QPP TG regression
DMN DeltaFC after QPP GS regression
DMN DeltaFC after QPP WT regression
DMN DeltaFC after QPP TG regression
DMN-TPN DeltaFC after QPP GS regression
DMN-TPN DeltaFC after QPP WT regression
DMN-TPN DeltaFC after QPP TG regression
all rsfMRI measures
DMN FC after GSR
DMN-TPN FC after GSR
DMN DeltaFC after GSR
DMN-TPN DeltaFC after GSR
after GSR
B
Absolute beta weights (100 cross-validations)
180
160
140
120
100
80
60
40
20
0
DMN FC
DMN -TPN FC
QPP GS
QPP WT
QPP TG
DMN FC after QPP GS regression
DMN FC after QPP WT regression
DMN FC after QPP TG regression
DMN-TPN FC after QPP GS regression
DMN-TPN FC after QPP WT regression
DMN-TPN FC after QPP TG regression
DMN DeltaFC after QPP GS regression
DMN DeltaFC after QPP WT regression
DMN DeltaFC after QPP TG regression
DMN-TPN DeltaFC after QPP GS regression
DMN-TPN DeltaFC after QPP WT regression
DMN-TPN DeltaFC after QPP TG regression
DMN FC after GSR
DMN-TPN FC after GSR
DMN DeltaFC after GSR
DMN-TPN DeltaFC after GSR
after GSR

Supplementary Figure 8. Network functional connectivity after global signal regression lowers classification performance

A) Upper panel displays the same mean AUC-values for all rsfMRI measures, as was shown in Fig. 6. Additionally, the four right bars display the AUC-values for DMN- and DMN-TPN-like FC measures after GSR. Statistical comparisons between the AUC-values are indicated in the matrix below. Red colour indicates $p<0.05$. The upper right side indicates uncorrected p-values, while the lower left bottom indicates p-values after Bonferroni post-hoc correction. Note that GSR-derived FC measures performed significantly less well than DMN-like FC prior to GSR. **B)** All absolute beta values for each individual measure in the combined model, which now also included rsfMRI measures after GSR. High beta-values imply the importance of the respective measure. DMN- and DMN-TPN-like ΔFC after QPP TG regression, together with DMN-TPN-like ΔFC after QPP WT regression, still showed the highest contributions. RsfMRI measures after GSR showed almost no contribution.

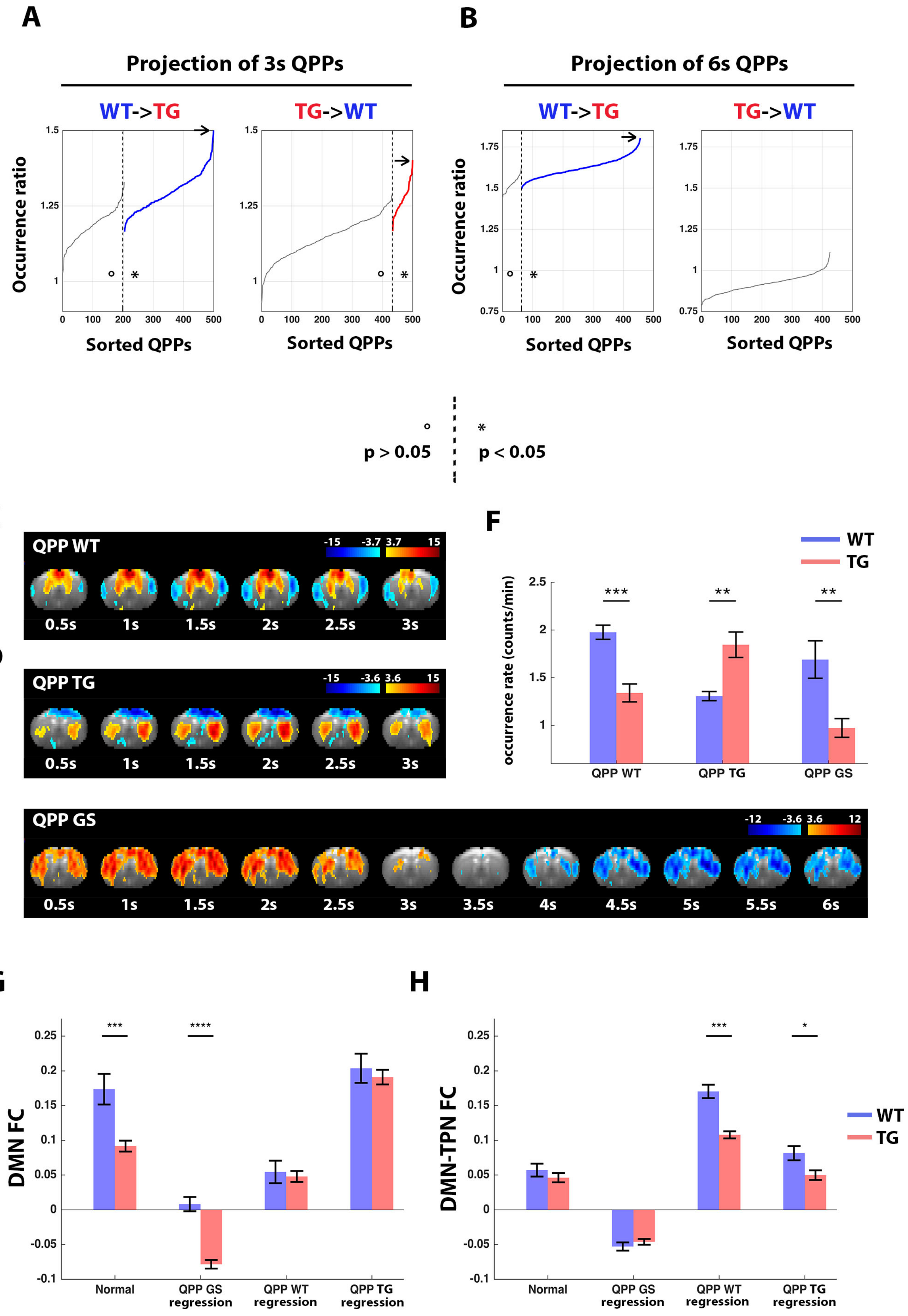
A
Projection of 3s QPPs
WT->TG
TG->WT
Occurrence ratio
Sorted QPPs
B
Projection of 6s QPPs
WT->TG
TG->WT
Occurrence ratio
Sorted QPPs
°
p > 0.05
*
p < 0.05
C
QPP WT
0.5s 1s 1.5s 2s 2.5s 3s
D
QPP TG
0.5s 1s 1.5s 2s 2.5s 3s
E
QPP GS
0.5s 1s 1.5s 2s 2.5s 3s 3.5s 4s 4.5s 5s 5.5s 6s
F
WT
TG
occurrence rate (counts/min)
QPP WT
QPP TG
QPP GS
G
DMN FC
Normal
QPP GS regression
QPP WT regression
QPP TG regression
H
DMN-TPN FC
Normal
QPP GS regression
QPP WT regression
QPP TG regression
WT
TG

Supplementary Figure 9. Quasi-Periodic pattern identification based on maximal ratios of STC versus pSTC occurrence rates

WT and TG QPPs were compared across groups**,** using pSTC (cfr. Fig. 1). Panels display the group mean ratio of each QPP's subject-average occurrences in the reference group over its subject-average occurrences in the target group. QPPs were hereby split into two significance groups and in each they were sorted by increasing ratios: non-significant group differences (two-sample T-test, $p>0.05$) are indicated in grey, while significant ones are color-coded in correspondence with the reference group. The ideology of this procedure is to determine QPPs that are the most dissimilar between groups with regard to their occurrence rate. **A)** At 3 s window lengths, WT QPPs displayed higher occurrence ratios versus TG, and vice versa. WT QPP ratios versus TG were more often significant compared to TG QPP ratios versus WT. **B)** At 6 s window lengths, WT QPPs displayed many significant and higher occurrence ratios versus TG. TG QPPs displayed mostly lower and non-significant ratios versus WT. A small set of high-contrast QPPs, similar to those observed in (A), were removed from the 6 s QPPs, based on a 95 % confidence interval of $\overline{\mathrm{QPP}Cv}$ (cfr. Supplementary Fig. 1). **C-E)** For further investigation, one QPP was selected from each group comparison, from the significant highest occurrence rate ratios (A-B, black arrows). A total of three QPPs were thus selected. Note their nearly identical spatiotemporal similarity to the QPPs presented in Fig. 2. **F)** Subject mean occurrence rates for all QPPs, based on STC and pSTC (two-sample T-test, $^{**}p<0.01$, $^{***}p<0.001$, error flags show standard error). **G-H)**. Similar as in Fig. 5, the effect of QPP regression was evaluated for DMN- and DMN-TPN-like FC. Note that the results are nearly identical. Overall this figure confirms that both selection criteria presented in the current study determine consistent results.

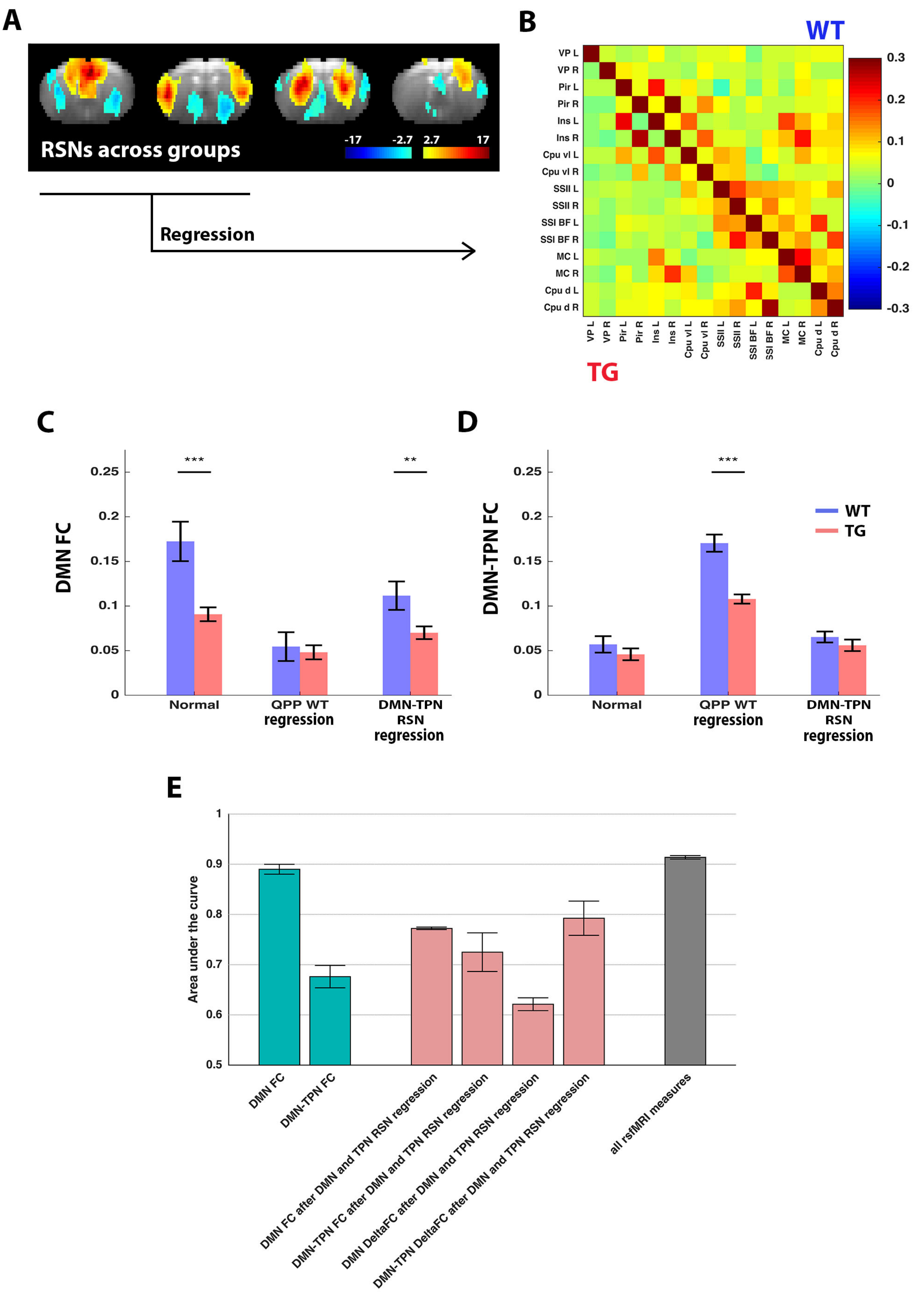
A
RSNs across groups
-17
-2.7
2.7
17
Regression
B
WT
TG
VP L
VP R
Pir L
Pir R
Ins L
Ins R
Cpu vl L
Cpu vl R
SSII L
SSII R
SSI BF L
SSI BF R
MC L
MC R
Cpu d L
Cpu d R
0.3
0.2
0.1
0
-0.1
-0.2
-0.3
C
DMN FC
***
**
0.25
0.2
0.15
0.1
0.05
0
Normal
QPP WT regression
DMN-TPN RSN regression
D
DMN-TPN FC
***
WT
TG
E
Area under the curve
1
0.9
0.8
0.7
0.6
0.5
DMN FC
DMN-TPN FC
DMN FC after DMN and TPN RSN regression
DMN-TPN FC after DMN and TPN RSN regression
DMN DeltaFC after DMN and TPN RSN regression
DMN-TPN DeltaFC after DMN and TPN RSN regression
all rsfMRI measures

Supplementary Figure 10. Classification based on resting state networks regression

A) ICA-derived RSNs for a combined analysis of WT and TG groups. The two left RSNs, which match DMN- and TPN-like networks, explained the highest percentage of variance compared to the other two RSNs, after regressing them out of all subject's image series. RSNs are displayed as thresholded T-maps, overlain on respective brain images (one-sample T-test, FDR $p<0.05$). **B)** ROI-based zFC matrix after combined regression of DMN- and TPN-like RSNs. The reasoning for regressing these two QPPs was to mimic spatiotemporal structure present in QPP WT. In the top triangle are shown the subject-average values for the WT group and in the lower triangle values for the TG group. Note there is little difference compared to the zFC matrix presented in Fig. 4A. **C-D)** DMN- and DMN-TPN-like FC under normal conditions (left), after regression of QPP WT (middle), and after regression of the DMN- and TPN-like RSNs. RSN regression reproduced a similar less strong effect on DMN FC reduction compared to QPP WT regression. No similar effect was observed for DMN-TPN-like FC. **E)** Classification analysis. Panel displays the same mean AUC-values for DMN- and DMN-TPN-like FC (left), as was shown in Fig. 6. Additionally, the four middle bars display the AUC-values for DMN- and DMN-TPN-like FC measures after RSN regression. The utter right bar display the AUC values for a combined model based on the 6 displayed parameters. Note that classification was not improved based on these parameters, contrasted by the increased classification based on QPP-derived FC measures (Fig. 6).
